# BMI and Deescalation From Ticagrelor to Clopidogrel in Patients With Acute Myocardial Infarction: A Post Hoc Analysis of the TALOS-AMI Trial

**DOI:** 10.1001/jamanetworkopen.2024.61916

**Published:** 2025-02-27

**Authors:** Seonghyeon Bu, Chan Joon Kim, Sungmin Lim, Jaehyuk Jang, Mahn-Won Park, Ik Jun Choi, Donggyu Moon, Byung-Hee Hwang, Eun Ho Choo, Kwan Yong Lee, Yun Seok Choi, Hee-Yeol Kim, Ki-Dong Yoo, Doo Soo Jeon, Kiyuk Chang

**Affiliations:** 1 Division of Cardiology, Department of Internal Medicine, Uijeongbu St Mary’s Hospital, College of Medicine, The Catholic University of Korea, Seoul, Republic of Korea; 2 Catholic Research Institute for Intractable Cardiovascular Disease, College of Medicine, The Catholic University of Korea, Seoul, Republic of Korea; 3 Division of Cardiology, Department of Internal Medicine, Daejeon St Mary’s Hospital, College of Medicine, The Catholic University of Korea, Seoul, Republic of Korea; 4 Division of Cardiology, Department of Internal Medicine, Incheon St Mary’s Hospital, College of Medicine, The Catholic University of Korea, Seoul, Republic of Korea; 5 Division of Cardiology, Department of Internal Medicine, St Vincent’s Hospital, College of Medicine, The Catholic University of Korea, Seoul, Republic of Korea; 6 Division of Cardiology, Department of Internal Medicine, Seoul St Mary’s Hospital, College of Medicine, The Catholic University of Korea, Seoul, Republic of Korea; 7 Division of Cardiology, Department of Internal Medicine, Bucheon St Mary’s Hospital, College of Medicine, The Catholic University of Korea, Seoul, Republic of Korea

## Abstract

**Importance:**

The potential benefits of P2Y12 inhibitor deescalation for acute myocardial infarction after percutaneous coronary intervention may be influenced by body mass index (BMI).

**Objectives:**

To investigate the association of BMI on deescalation outcomes after 12 months in patients with acute myocardial infarction after percutaneous coronary intervention who were initially treated with aspirin plus ticagrelor for 1 month, and to assess whether BMI-based switching from aspirin plus ticagrelor (active control strategy) to aspirin plus clopidogrel (deescalation strategy) is associated with individualized benefits.

**Design, Setting, and Participants:**

This study is a post hoc analysis, based on BMI, of data from the TALOS-AMI (Ticagrelor vs Clopidogrel in Stabilized Patients with Acute Myocardial Infarction) randomized clinical trial. Data were collected from February 14, 2014, to December 31, 2018, with follow-up to January 21, 2021. Analyses were conducted from December 1, 2021, to August 21, 2024. Among 2697 trial participants from 32 centers in South Korea, 2686 participants whose BMI data were available were included.

**Exposure:**

All patients received aspirin plus ticagrelor for 1 month after percutaneous coronary intervention. Stabilized patients were then randomized to either the active control or deescalation strategy for an additional 11 months.

**Main Outcomes and Measures:**

The primary end point was a composite of cardiovascular death, myocardial infarction, stroke, and Bleeding Academic Research Consortium bleeding type 2, 3, or 5 at 12 months after percutaneous coronary intervention. The trial compared the active control and deescalation strategies according to BMIs, including an interaction test.

**Results:**

Of the 2686 patients included (mean [SD] age, 60.0 [11.4] years; 2234 [83.2%] male), 2344 (1161 in the deescalation group and 1183 in the active control group) had a BMI less than 28, and 342 (184 in the deescalation group and 158 in the active control group) had a BMI of 28 or greater. The deescalation strategy was associated with significantly reduced composite outcomes compared with the active control strategy in the group with a BMI less than 28 (53 [4.6%] vs 98 [8.3%]; adjusted hazard ratio, 0.54; 95% CI, 0.39-0.76; *P* < .001), primarily due to fewer bleeding complications. There was no association in the group with a BMI of 28 or greater (6 [3.3%] vs 5 [3.2%]; adjusted hazard ratio, 1.07; 95% CI, 0.33-3.50; *P* = .91).

**Conclusions and Relevance:**

In this post hoc analysis of the TALOS-AMI randomized clinical trial, in stabilized patients with acute myocardial infarction, an unguided deescalation strategy of switching from ticagrelor to clopidogrel after 1 month was associated with better clinical outcomes in those with lower BMIs.

**Trial Registration:**

ClinicalTrials.gov Identifier: NCT02018055

## Key Points

**Question:**

Is body mass index (BMI) associated with P2Y12 inhibitor deescalation outcomes in patients with acute myocardial infarction after percutaneous coronary intervention?

**Findings:**

In this post hoc analysis of 2686 participants in the TALOS-AMI randomized clinical trial, deescalating from aspirin plus ticagrelor to aspirin plus clopidogrel at 1 month post intervention was associated with significantly reduced composite outcomes compared with an active control strategy in the lower BMI group but not in the higher BMI group, primarily due to fewer bleeding complications.

**Meaning:**

These findings suggest that a deescalation strategy may be beneficial in patients with stabilized acute myocardial infarction with lower BMI but not those with higher BMI.

## Teaser

This post hoc analysis of a randomized clinical trial examines the association of body mass index (BMI) with drug deescalation outcomes in patients with acute myocardial infarction (AMI) after percutaneous coronary intervention.

## Introduction

Cardiovascular disease is the leading cause of mortality worldwide, with acute myocardial infarction (AMI) contributing substantially to this morbidity.^1^ Percutaneous coronary intervention (PCI) followed by antiplatelet therapy has markedly improved outcomes for patients with AMI. Recently, potent P2Y12 inhibitors, ticagrelor or prasugrel, have been developed as standard therapies for AMI.^2,3^ However, the optimal P2Y12 inhibitor therapy after PCI remains uncertain due to bleeding.

The Ticagrelor vs Clopidogrel in Stabilized Patients with Acute Myocardial Infarction (TALOS-AMI) trial showed better clinical outcomes in stabilized patients with AMI who underwent PCI and were administered aspirin plus ticagrelor for 1 month and then switched to aspirin plus clopidogrel than in those who were taking aspirin plus continuous ticagrelor.^4^ The observed results were primarily attributed to reduced bleeding events. The obesity paradox indicates higher mortality in patients with acute coronary syndrome who underwent PCI with lower body mass index (BMI) than in those with higher BMI. Meanwhile, the East Asian paradox indicates increased bleeding risk in East Asian patients than in White patients. Despite ongoing debate, these phenomena appear to be associated with an increased bleeding risk according to lower BMI or body weight. However, the association between BMI and optimal P2Y12 inhibitors therapy after PCI, which increases the bleeding risk, remains unclear. Therefore, we conducted a post hoc analysis of the TALOS-AMI data to examine the association between BMI and the outcomes of deescalation strategies of switching from aspirin plus ticagrelor to aspirin plus clopidogrel.

## Methods

### Study Design

The design, inclusion and exclusion criteria, and outcomes of the TALOS-AMI trial have been published previously.^5^ The TALOS-AMI trial was a randomized, noninferiority study that included patients with AMI from 32 sites in South Korea. All enrolled patients underwent PCI and received aspirin plus ticagrelor for 1 month after PCI. Stabilized patients without ischemic or severe bleeding complications (n = 2697) were randomly assigned to the following 2 groups at the end of 1 month: aspirin plus clopidogrel (deescalation group, n = 1349) or aspirin plus ticagrelor (active control group, n = 1348). For 12 months after PCI, the patients were followed up by office visits or telephone contact. Data were collected between February 14, 2014, and December 31, 2018, with follow-up to January 21, 2021. This study was approved by the institutional review boards at each participating institution and adhered to the principles of the Declaration of Helsinki.^6^ All participants provided written informed consent. This study followed the Consolidated Standards of Reporting Trials (CONSORT) reporting guideline. The trial protocol is available in Supplement 1.

**Figure 1.**
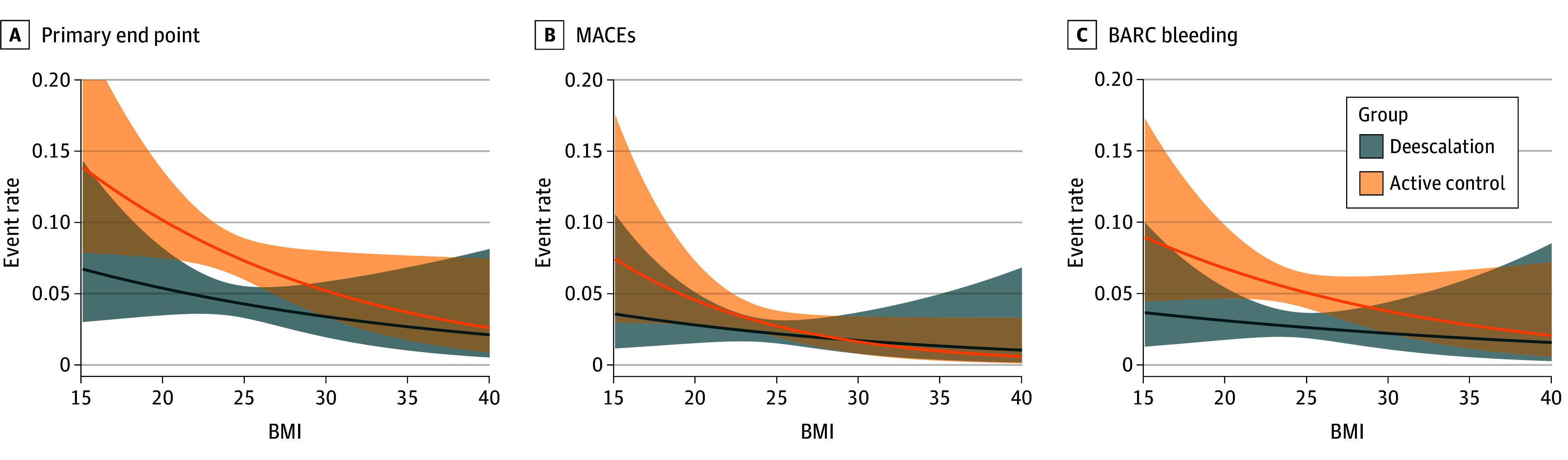
Spline Curve of the Event Rates The primary outcome was a composite of cardiovascular death, myocardial infarction, stroke, and Bleeding Academic Research Consortium (BARC) bleeding type 2, 3, or 5 from 1 to 12 months after percutaneous coronary intervention. Major adverse cardiac event (MACE) is defined as the composite of cardiovascular death, stroke, and myocardial infarction. BARC bleeding comprises bleeding event type 2, 3, or 5 according to the BARC criteria. BMI indicates body mass index (calculated as weight in kilograms divided by height in meters squared).

The present study was designed to assess the association between the outcomes of the deescalation strategy and the baseline BMI (calculated as weight in kilograms divided by height in meters squared) of patients from the original TALOS-AMI trial. To divide patients into groups by BMI, the threshold was determined as follows. We plotted spline curves of event rate according to continuous BMI (Figure 1) and found that the difference between primary outcomes of 2 spline curves linearly decreased as BMI increased. The Subpopulation Treatment Effect Pattern Plot (STEPP) method, a statistical technique to identify thresholds where the treatment may be more effective, was used to verify the threshold for distinguishing patients with varying clinical efficacies of deescalation, confirming the BMI threshold value of 28.

### Outcomes

As in the original TALOS-AMI study, the primary outcome for the present study was the occurrence of major adverse cardiovascular events (MACEs), a composite of cardiovascular death, myocardial infarction, stroke, and bleeding type 2, 3, or 5 according to the Bleeding Academic Research Consortium (BARC) criteria, 12 months after the index PCI. The main secondary outcomes included a composite of cardiovascular death, myocardial infarction, and stroke, a composite of BARC bleeding type 2, 3, or 5, and the individual BARC bleeding components. Other secondary outcomes included individual components of the primary outcome, all-cause death, ischemia-driven revascularization, and stent thrombosis.

### Statistical Analysis

Data were analyzed between December 1, 2021, and August 21, 2024. Baseline characteristics are expressed as means (SDs) or numbers (percentages). Continuous variables were compared using the independent-sample, 2-tailed *t* test or Wilcoxon rank-sum test based on the normality of distributions, and categorical variables were compared using the χ^2^ test. We used a Cox proportional hazards regression model to evaluate the association between different treatment and outcome measures (MACEs and bleeding), adjusted for age and sex, including an interaction test. Testing for trends was performed to verify the association between different treatment and outcome measures. All statistical analyses were performed using SAS software, version 9.4 (SAS Institute Inc) and R software, version 4.2.2 (R Foundation). A 2-sided *P* < .05 was considered statistically significant.

## Results

Of the 2697 participants from 32 centers in South Korea who were enrolled in the TALOS-AMI trial, 2686 patients whose BMI data were available were included (mean [SD] age, 60.0 [11.4] years; 452 [16.8%] female and 2234 [83.2%] male). The participants were stratified into groups with BMI less than 28 or BMI of 28 or greater. Each group was further subdivided into the deescalation (aspirin plus clopidogrel) and active control (aspirin plus ticagrelor) subgroups. The deescalation and active control subgroups included 1161 and 1183 patients with BMIs less than 28, respectively, and 184 and 158 patients with BMIs of 28 or greater, respectively (eFigure in Supplement 2).

In the group with BMI less than 28, the deescalation and active control subgroups did not significantly differ in their demographic and procedural characteristics (Table 1; eTable 1 in Supplement 2). Differences were observed in smoking, estimated glomerular filtration rate, and stroke history among patients with BMI of 28 or greater.

**Table 1.** Baseline Characteristics of Patients According to BMI

Characteristic	BMI <28 (n = 2344)			BMI ≥28 (n = 342)			*P* value for difference
	Deescalation, No. (%) (n = 1161)^a^	Active control, No. (%) (n = 1183)^b^	*P* value	Deescalation, No. (%) (n = 184)^a^	Active control, No. (%) (n = 158)^b^	*P* value	
Age, mean (SD), y	60.90 (10.90)	60.83 (11.12)	.88	54.91 (12.15)	52.87 (11.42)	.11	<.001
Age >75 y	141 (12.1)	157 (13.3)	.41	16 (8.7)	7 (4.4)	.12	<.001
Sex							
Male	974 (83.9)	969 (81.9)	.20	155 (84.2)	136 (86.1)	.64	.31
Female	187 (16.1)	214 (18.1)		29 (15.8)	22 (13.9)		
CVD risk factor							
Hypertension	536 (46.2)	572 (48.4)	.29	117 (63.6)	87 (55.1)	.11	<.001
Diabetes	301 (25.9)	322 (27.2)	.48	59 (32.1)	45 (28.5)	.47	.14
Insulin	22 (1.9)	23 (1.9)	.93	6 (3.3)	5 (3.2)	.96	.12
Dyslipidemia	474 (40.8)	472 (39.9)	.65	88 (47.8)	81 (51.3)	.53	<.001
Smoking							
Nonsmoker	385 (33.2)	406 (34.3)	.82	72 (39.1)	32 (20.3)	<.001	.03
Former	201 (17.3)	205 (17.3)		20 (10.9)	26 (16.5)		
Current	575 (49.5)	572 (48.4)		92 (50.0)	100 (63.3)		
Impaired kidney function^c^							
eGFR, mean (SD), mL/min	78.74 (25.43)	80.12 (27.83)	.21	102.19 (30.61)	111.96 (38.95)	.01	<.001
eGFR <60 mL/min/1.73 m^2^	280 (24.1)	277 (23.4)	.69	24 (13.0)	9 (5.7)	.02	<.001
Past medical history							
PCI	50 (4.3)	54 (4.6)	.76	11 (6.0)	6 (3.8)	.36	.66
CABG	0	2 (0.2)	.25	1 (0.5)	1 (0.6)	>.99	.08
CVA	41 (3.5)	48 (4.1)	.50	12 (6.5)	2 (1.3)	.02	.79
Clinical presentation							
STEMI	505 (43.5)	538 (45.5)	.34	76 (41.3)	73 (46.2)	.36	<.001
NSTEMI	656 (56.5)	645 (54.5)		108 (58.7)	85 (53.8)		
LVEF <40%	90 (7.9)	88 (7.7)	.84	13 (7.2)	5 (3.3)	.13	.13

Abbreviations: BMI, body mass index (calculated as weight in kilograms divided by height in meters squared); CABG, coronary artery bypass surgery; CVA, cerebrovascular accident; CVD, cardiovascular disease; eGFR, estimated glomerular filtration rate; PCI, percutaneous coronary intervention; NSTEMI, non–ST-segment elevation myocardial infarction; STEMI, ST-segment elevation myocardial infarction.

^a^ The deescalation group was administered aspirin plus clopidogrel.

^b^ The active control group administered aspirin plus ticagrelor.

^c^ Impaired kidney function was defined as an eGFR of less than 60 mL/min/1.73 m^2^ of body surface area at presentation.

The spline curve revealed that the event rates for the primary outcome decreased with increasing BMI, and similar shapes were observed for the spline curves of the event rates for MACEs and BARC bleeding (Figure 1). In addition, the differences between primary outcomes of 2 spline curves linearly decreased as BMI increased (adjusted *P* = .045 for trend) (eTable 2 in Supplement 2).

**Figure 2.**
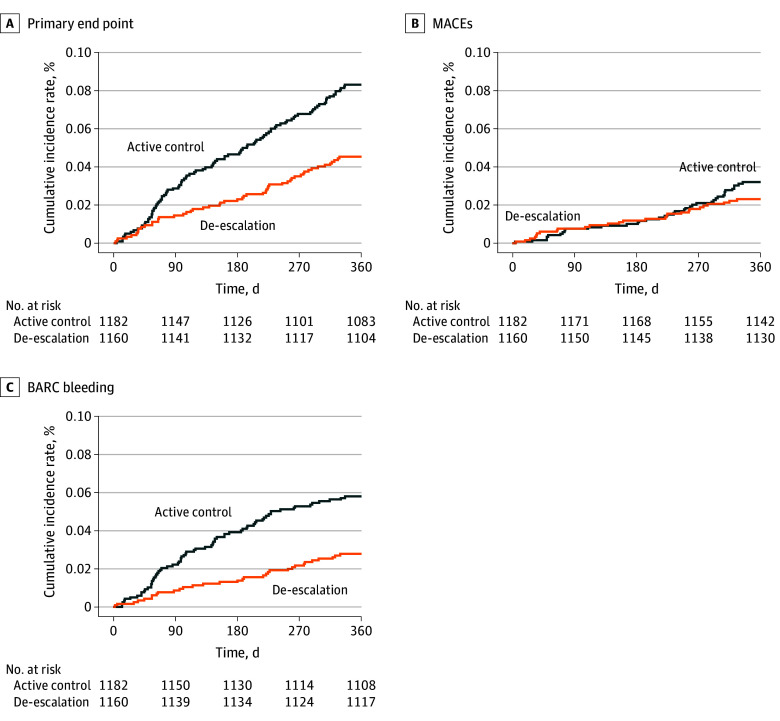
Kaplan-Meier Estimates for Patients With Body Mass Index Less Than 28 The primary outcome was a composite of cardiovascular death, myocardial infarction, stroke, and Bleeding Academic Research Consortium (BARC) bleeding type 2, 3, or 5 from 1 to 12 months after percutaneous coronary intervention. Major adverse cardiac event (MACE) is defined as the composite of cardiovascular death, stroke, and myocardial infarction. BARC bleeding comprises bleeding event type 2, 3, or 5 according to the BARC criteria.

**Figure 3.**
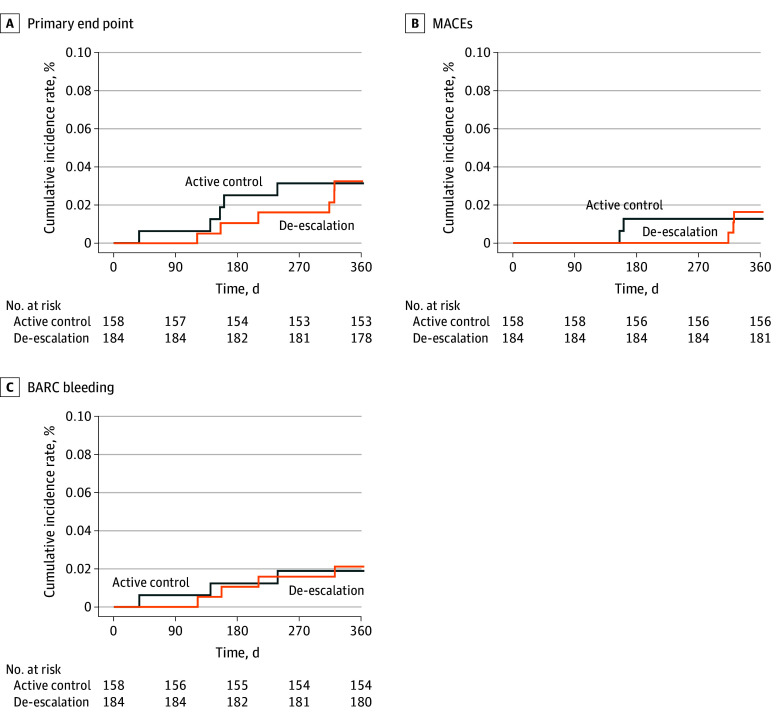
Kaplan-Meier Estimates for Patients With Body Mass Index of 28 or Greater The primary outcome was a composite of cardiovascular death, myocardial infarction, stroke, and Bleeding Academic Research Consortium (BARC) bleeding type 2, 3, or 5 from 1 to 12 months after percutaneous coronary intervention. Major adverse cardiac event (MACE) is defined as the composite of cardiovascular death, stroke, and myocardial infarction. BARC bleeding comprises bleeding event type 2, 3, or 5 according to the BARC criteria.

In the group with BMI less than 28, the primary outcome (ie, a composite of cardiovascular death, myocardial infarction, stroke, and BARC bleeding types 2, 3, and 5) was significantly less frequent in the deescalation subgroup than in the active control subgroup (53 [4.6%] vs 98 [8.3%]; adjusted hazard ratio [AHR], 0.54; 95% CI, 0.39-0.76). No significant difference was observed in MACE incidence between the deescalation and active control subgroups (27 [2.3%] vs 38 [3.2%]; AHR, 0.72; 95% CI, 0.44-1.18). The incidence of BARC bleeding type 2, 3, and 5 events was significantly lower in the deescalation subgroup than in the active control subgroup (32 [2.8%] vs 68 [5.8%]; AHR, 0.47; 95% CI, 0.31-0.72), and the incidence of each component of BARC bleeding events was significantly lower in the deescalation group than in the active control group (BARC bleeding type 2: 24 [2.1%] vs 47 [4.0%]; AHR, 0.51; 95% CI, 0.31-0.84; type 3: 14 [1.2%] vs 28 [2.4%]; AHR, 0.51; 95% CI, 0.27-0.97; types 3 and 5: 14 [1.2%] vs 28 [2.4%]; AHR, 0.51; 95% CI, 0.27-0.97) (Table 2 and Figure 2). In the group with BMI of 28 or higher, the deescalation and active control subgroups did not significantly differ in the primary outcomes (6 [3.3%] vs 5 [3.2%]; AHR, 1.07; 95% CI, 0.33-3.50), MACEs (3 [1.6%] vs 2 [1.3%]; AHR, 1.33; 95% CI, 0.22-7.95), and BARC 2, 3, and 5 bleeding (4 [2.2%] vs 3 [1.9%]; AHR, 1.19; 95% CI, 0.27-5.31) (Table 2 and Figure 3). The risk reduction for deescalation in patients with BMIs less than 28 was 46%, which was similar to the 45% risk reduction for deescalation in the overall cohort, including patients with BMI across the spectrum referenced in the original TALOS-AMI trial. However, the risk due to deescalation tended to increase by 7% in patients with BMIs of 28 or greater.

**Table 2.** Primary Outcomes

Outcome	BMI <28 (n = 2344)						BMI ≥28 (n = 342)						*P* value for interaction	*P* value for interaction (adjusted)
	Deescalation, No. (%) (n = 1161)	Active control, No. (%) (n = 1183)	Crude HR	*P* value	Adjusted HR^a^	*P* value	De-scalation, No. (%) (n = 184)	Active control, No. (%) (n = 158)	Crude HR	*P* value	Adjusted HR^a^	*P* value		
Primary end point	53 (4.6)	98 (8.3)	0.54 (0.39-0.76)	<.001	0.54 (0.39-0.76)	<.001	6 (3.3)	5 (3.2)	1.02 (0.31-3.35)	.97	1.07 (0.33-3.50)	.91	.31	.33
MACE	27 (2.3)	38 (3.2)	0.72 (0.44-1.18)	.20	0.72 (0.44-1.18)	.19	3 (1.6)	2 (1.3)	1.28 (0.22-7.67)	.79	1.33 (0.22-7.95)	.76	.55	.59
BARC bleeding														
Type 2, 3, 5	32 (2.8)	68 (5.8)	0.47 (0.31-0.72)	<.001	0.47 (0.31-0.72)	<.001	4 (2.2)	3 (1.9)	1.13 (0.25-5.07)	.87	1.19 (0.27-5.31)	.82	.27	.27
Type 3, 5	14 (1.2)	28 (2.4)	0.51 (0.27-0.96)	.04	0.51 (0.27-0.97)	.04	1 (0.5)	0	2.67 (0.03-258)	.67	3.75 (0.03-568.9)	.61	.99	.99
Type 2	24 (2.1)	47 (4.0)	0.52 (0.32-0.84)	.01	0.51 (0.31-0.84)	.01	3 (1.6)	3 (1.9)	0.86 (0.17-4.24)	.85	0.90 (0.18-4.44)	.89	.56	.55
Type 3	14 (1.2)	28 (2.4)	0.51 (0.27-0.96)	.04	0.51 (0.27-0.97)	.04	1 (0.5)	0	2.67 (0.03-258.0)	.67	3.75 (0.03-568.9)	.61	.99	.99
Type 5	1 (0.1)	NA	NA	NA	NA	NA	0	NA	NA	NA	NA	NA	NA	NA

Abbreviations: BARC, Bleeding Academic Research Consortium; BMI, body mass index (calculated as weight in kilograms divided by height in meters squared); HR, hazard ratio; MACE, major adverse cardiovascular event; NA, not applicable.

^a^ Adjusted for age (>75 years) and sex.

Regarding other secondary outcomes, no significant differences in all-cause death (11 [1.0%] vs 9 [0.8%]; AHR, 1.28; 95% CI, 0.53-3.09 in the BMI <28 group and 0 [0%] vs 1 [0.6%]; AHR, 0.25; 95% CI, 0.00-22.73 in the BMI ≥28 group), myocardial infarction (9 [0.8%] vs 20 [1.7%]; AHR, 0.46; 95% CI, 0.21-1.01 and 3 [1.6%] vs 0 [0%]; AHR, 6.23; 95% CI, 0.19-208.00), ischemia-driven revascularization (12 [1.0%] vs 15 [1.3%]; AHR, 0.79; 95% CI, 0.37-1.69 and 5 [2.7%] vs 2 [1.3%]; AHR, 2.06; 95% CI, 0.40-10.66), and stent thrombosis (1 [0.1%] vs 3 [0.3%]; AHR, 0.44; 95% CI, 0.05-3.71 and 2 [1.1%] vs 0 [0%]; AHR, 4.32; 95% CI, 0.10-197.00) were observed between the deescalation and active control subgroups in both the BMI groups (eTable 3 in Supplement 2).

The use of a BMI cutoff of 28 in this study was derived from statistical analyses, including the STEPP method and spline curve, between BMI and the deescalation strategy. When using the World Health Organization definition of obesity as a BMI of 25, some results were inconsistent: for the deescalation subpopulation, the AHR was 0.51 (95% CI, 0.34-0.77) in the group with a BMI less than 25 and was 0.62 (95% CI, 0.37-1.03) in those with a BMI of 25 or greater (eTable 4 in Supplement 2).

## Discussion

This study examined the association between BMI and the clinical outcomes of different antiplatelet regimens. Our findings suggest that stabilized patients with AMI and a BMI less than 28 may benefit significantly from deescalation by switching from aspirin plus ticagrelor to aspirin plus clopidogrel after 1 month from index PCI, mainly due to a reduction in bleeding events. However, such benefits were not observed in patients with a BMI of 28 or greater.

This differential response to antiplatelet therapy based on BMI may be attributed to the distinct pharmacodynamic and pharmacokinetic properties of these drugs in patients with different BMIs, potentially influenced by altered distribution volumes, metabolism rates, and drug clearance. Regarding clopidogrel, a study on the relationship between body weight and the concentrations of active metabolites revealed that the area under the concentration curve was 1.4 times higher in patients with low body weight (<60 kg) than in those with high body weight (≥60 kg), which was significant.^7^

BMI is also included in scoring systems used for bleeding risk assessment. The PARIS (Patterns of Nonadherence to Antiplatelet Regimen in Stented Patients) score, designed to evaluate the risk of major bleeding during a 2-year period after PCI with dual antiplatelet therapy, includes lower BMI as a parameter associated with an increased risk of bleeding.^8^ Similarly, the National Cardiovascular Disease Registry CathPCI Bleeding Risk Score, which focuses on predicting bleeding within 3 days after PCI, includes lower BMI as a parameter that increases the bleeding risk.^9^ The TRITON-TIMI 38 (Trial to Assess Improvement in Therapeutic Outcomes by Optimizing Platelet Inhibition With Prasugrel–Thrombolysis In Myocardial Infarction 38) study did not recommend the use of prasugrel in patients weighing less than 60 kg because higher levels of active prasugrel metabolites were expected to increase the risk of bleeding.^10^ The BMI-stratified subgroup analysis in the TROPICAL-ACS trial (Testing Responsiveness to Platelet Inhibition on Chronic Antiplatelet Treatment for Acute Coronary Syndromes) also showed different outcomes based on BMI; patients with a BMI below 25 had significantly improved primary outcomes with a platelet function test–guided deescalation strategy of switching from prasugrel to clopidogrel. This change resulted in reduced bleeding events without a concurrent increase in ischemic events. In contrast, for patients with a BMI above 25, this deescalation strategy resulted in outcomes similar to those for patients who received the standard continuous prasugrel treatment.^11^ The BMI subgroup analysis in the GLOBAL LEADERS trial examined the effects of BMI on clinical outcomes by comparing patients receiving ticagrelor monotherapy after 1 month of dual antiplatelet therapy with ticagrelor and those receiving standard therapy. Patients with a BMI below 27 and acute coronary syndrome showed significantly improved primary outcomes regarding all-cause mortality and Q-wave myocardial infarction; however, this improvement was not observed in patients with a BMI of 27 or greater.^12^ The HOST-EXAM (Harmonizing Optimal Strategy for Treatment of Coronary Artery Stenosis–Extended Antiplatelet Monotherapy) post hoc study investigated the effects of BMI on the composite outcome of all-cause death, nonfatal myocardial infarction, stroke, readmission due to acute coronary syndrome, and major bleeding of BARC type 3 or higher. The study examined patients receiving long-term antiplatelet monotherapy after at least 6 months from PCI and showed the relationship between a lower BMI (<25) and a higher risk of primary composite outcomes, which was driven by increased incidences of bleeding events.^13^ These results might be explained by the East Asian paradox, which states that the risk of bleeding is higher in East Asian populations than in Western populations. One of the reasons for this phenomenon might be that East Asian individuals tend to have a lower body weight.^14,15^ According to the concept of the obesity paradox, the mortality rate after PCI tends to be lower in patients with a higher BMI,^16,17,18,19^ which is related to increased bleeding risks after the use of antiplatelet agents in patients with lower BMIs.

Several studies have found that bleeding rates were not significantly higher with ticagrelor than with clopidogrel across BMI without heterogeneity.^20,21^ These results could be interpreted as meaning that there was no significant difference in bleeding risk between ticagrelor and clopidogrel in patients with a higher BMI. In addition, the message of our analysis is that in patients with stabilized AMI, the bleeding rates were higher with ticagrelor than with clopidogrel when the BMI was low.

Although this study examined the deescalation strategy of unguided switching from ticagrelor to clopidogrel, the deescalation strategy of reducing the dose from 90 mg to 60 mg of ticagrelor might be an alternative strategy. Studies have shown that bleeding events were numerically lower in the 60-mg group than in the 90-mg group, and the mean plasma ticagrelor level 2 hours after administration in the 60-mg group was two-thirds that of the 90-mg group.^22,23^ Additional studies are warranted to examine whether the dose deescalation strategy has comparable efficacy to the deescalation strategy of switching to clopidogrel based on BMI.

### Limitations

This study has some limitations. First, because this study was a post hoc analysis of the TALOS-AMI trial and not prespecified, the applicability of the findings may be constrained, given that the study population may not be representative of all patients undergoing PCI for AMI. Second, we only analyzed the BMI of patients at baseline and did not assess potential changes in BMI during the follow-up period. Third, the group with a BMI of 28 or greater was relatively small, which might make it challenging to support the threshold value of 28. Although the dichotomy of BMI by the STEPP method was used to show the impact of deescalation treatment based on BMI, 28 is not the absolute threshold value. Fourth, all patients in this study were enrolled in South Korea. Because Asian patients have lower BMIs on average compared with other ethnic groups, this may limit generalizability of the findings.

### Conclusions

Findings from this post hoc analysis of the TALOS-AMI study suggest that the outcomes associated with deescalation strategies vary by BMI, with the greatest impact of deescalation being seen in those with lower BMIs. The BMI threshold of 28 should be cautiously interpreted.

## Notes

Supplement 1.
Trial Protocol and Statistical Analysis Plan

Supplement 2. **eTable 1.** Procedural Characteristics According to BMI **eTable 2.** Trend Analysis for BMI as a Continuous Variable **eTable 3.** Secondary Outcomes **eTable 4.** Primary Outcomes Based on BMI 25 kg/m^2^ **eFigure.** Consort Diagram

Supplement 3.
Nonauthor Collaborators

Supplement 4.
Data Sharing Statement
