# Error in Figure 1

**DOI:** 10.1001/jamanetworkopen.2025.8167

**Published:** 2025-04-01

In the Original Investigation titled “BMI and Deescalation From Ticagrelor to Clopidogrel in Patients With Acute Myocardial Infarction: A Post Hoc Analysis of the TALOS-AMI Trial,”^1^ published on February 27, 2025, the color key labels in Figure 1 were swapped; the blue is meant to represent the deescalation group, and the orange represents the active control group. This article has been corrected.^1^
