# Supplementary material for: BMI and Deescalation From Ticagrelor to Clopidogrel in Patients With Acute Myocardial Infarction: A Post Hoc Analysis of the TALOS-AMI Trial
Source: JAMA Netw Open. 2025 Feb 27;8(2):e2461916. doi: 10.1001/jamanetworkopen.2024.61916 (PMC11868972; doi:10.1001/jamanetworkopen.2024.61916)
Supplement: Supplement 1. — Trial Protocol and Statistical Analysis Plan [file jamanetwopen-e2461916-s001.pdf]

A Prospective, multicenter, Randomized, Open-label Trial to Compare Efficacy and Safety of Clopidogrel vs Ticagrelor in stabilized Patients with Acute Myocardial Infarction after Percutaneous Coronary Intervention; **T**ic**A**grelor versus C**LO**pidogrel in **S**tabilized patients with **A**cute **M**yocardial **I**nfarction: TALOS-AMI

Protocol No.: TALOS-AMI

Protocol Version: 7.0

Development date: 2018.06.18

Confidentiality Agreement

Information in this study protocol is for investigators, clinical research coordinators, pharmacists, related administrative officers and IRB staffs of participating institutions. The following clinical trial protocol can be used only for the purpose of conducting and evaluating clinical trials and cannot be disclosed to any unrelated parties. Confidentiality should be strictly kept.

Confirmation of Clinical Trial Protocol Review

Investigator's Signature:

I have reviewed the contents of this protocol thoroughly, and hereby confirm that the protocol is designed to verify the characteristics of the test drug and does not raise ethical concerns. I agree that the clinical trial should proceed according to the KGCP (Korea Good Clinical Practice) Standard and accept the principles of the Declaration of Helsinki. I also approve the provision of research data and regular monitoring, am prepared for audit, and inspection, and agree to keep strict confidentiality.

Title : Principal Investigator

Kiyuk Chang

Printed Name	*signature*	*Date(YYYY/MM/DD)*

Table of Contents

Confirmation of Clinical Trial Protocol Review 1
Version History
PROTOCOL SUMMARY 5
DEFINITION 13
TIME TABLE 14
Title and Phase of Clinical Trial 16
1. Study Institution 16
2. Principal investigator, Sub-investigator and Clinical Research Cordinator 16
2.1. Principal Investigator 16
2.2. Sub-investigator and Clinical Researh Cordinator 16
3. Sponsor 16
4. Background and Objective 16
5. Study Drug 19
6. Study Disease 20
7. Subject Inclusion/Exclusion Criteria and Study Population 21
7.1. Subject Inclusion Criteria 21
7.2. Subject Exclusion Criteria 21
7.3. Study Population 22
8. Study Duration 23
9. Study Method 23
9.1. Study Process 24
9.2. Study Design 24
9.3. Randomization 24
9.4. Dosage and Method 25
9.5. Switching protocol (ticagreor to clopidogrel) 25
9.6. Combination treatment and cautions 26
10. Time table, clinical and laboratory measurement 27
10.1. Time table 27
10.2. Clinical and laboratory measurement 28
10.3. Visit schedule and assessment 35
11. Precautions and Expected Side Effects 37
11.1. Clopidogrel 37

11.2. Ticagrelor 37
12. Withdrawl of conent or Loss of Follow-up……...38
13. Event adjudication and reporting...38
14. Statistical Analysis...39
14.1. General Principal of Statistical Analysis...39
14.2.Evaluation standard...40
14.3. Evaluation Method...41
15. Safety (including side-effects) Evaluation Standard, Method and Reporting.43
15.1. Safety Related Definitions...43
15.2. Safety Evaluation Method 43
15.3. Reporting of Adverse Event and Serious adverse Event............................45
15.4. Safety Reporting..45
16. Informed Consent...46
17. Follow-up Treatment Post Clinical Trial...46
18. Subject Safety and Protective Measures…………………………………….47
18.1. Subject Safety and Protective Measures...47
18.2. Confidentiality..47
19. Requirements for Scientific Clinical Test……………………………………47
19.1. Protocol Deviation...47
19.2. Record Retention...48
19.3. Clinical Test Institution Monitoring..48
19.4. Investigator Responsibility..48
20. Study organization…..49
20.1. Steering Committe...49
20.2 Data Safety Monitoring Board (DSMB).. 49
20.3 Clinical Events Adjudication Committe...50
20.4. Data Coordination.. .50
21. Reference...50

Appendix 1) Study Institution and Personnel

Version History

Version	Summary of Changes	Authors
1.0	Initial release	Kiyuk Chang, Chan Joon Kim, Mahn-Won Park
2.0	Extension of the clinical trial period	Kiyuk Chang, Chan Joon Kim, Mahn-Won Park
3.0	Addition of the prescription details of in-hospital medication	Kiyuk Chang, Chan Joon Kim, Mahn-Won Park
4.0	Addition of a new institution as a clinical research institute	Kiyuk Chang, Chan Joon Kim, Mahn-Won Park
5.0	Refusal of genetic testing by one institution	Kiyuk Chang, Chan Joon Kim, Mahn-Won Park
6.0	Description of the change in the sample size	Kiyuk Chang, Chan Joon Kim, Mahn-Won Park
7.0	Extension of the clinical trial period	Kiyuk Chang, Chan Joon Kim, Mahn-Won Park

PROTOCOL SUMMARY

**Title**	A Prospective, multicenter, Randomized, Open-label Trial to Compare Efficacy and Safety of Clopidogrel vs Ticagrelor in stabilized Patients with Acute Myocardial Infarction (AMI) after Percutaneous Coronary Intervention (PCI); **T**ic**A**grelor versus **CLO**pidogrel in Stabilized patients with **A**cute **M**yocardial **I**nfarction: **TALOS-AMI**
**Principal investigator**	Dr. Kiyuk Chang, Division of Cardiology, Department of Internal Medicine, Seoul St. Mary's Hospital, The Catholic University of Korea<br><Appendix 1><br>*Clinical Trial Investigator (CI)
**Institution**	Appendix 1
**Study phase**	4
**Study design**	Prospective, multi-center, randomized, open trial
**Study Objective**	To investigate the efficacy and safety of switching from ticagrelor to clopidogrel in stabilized patients with AMI with no adverse events during the first month after an index PCI
**Study Drug**	Test drug: Clopidogrel (Pregrel)<br>Control drug: Ticagrelor (Brilinta)
**Study Duration**	Institutional Review Board approval (Oct. 17th, 2013 to Dec. 31st, 2020)
**Study Disease**	Acute myocardial infarction: ST-segment elevation MI (STEMI) and non-ST-segment elevation MI (NSTEMI)
**Study Population**	2590 (loss to follow-up: 10 %)<br>• Test group: 1295<br>• Control group: 1295
**Subject Inclusion & Exclusion Criteria**	**<u>Inclusion Criteria</u>**<br>1) Age ≥ 18 years<br>2) Patients with AMI (STEMI or NSTEMI) who are administered ticagrelor for 30 days after successful PCI with newer-generation drug eluting stents (DES)<br>*Definition of AMI follows the 3rd Universal Definition of MI.<br>3) Female patients with childbearing potential who agree to mandatory

	pregnancy test and have committed to using adequate contraception<br>4) Subjects who agree to the study protocol and the schedule of clinical follow-up, and provides informed, written consent, as approved by the appropriate IRB of the respective institution<br><br>**Exclusion Criteria**<br>1) Cardiogenic shock<br>2) Active internal bleeding, bleeding diathesis, or coagulopathy<br>3) Gastrointestinal bleeding or genitourinary bleeding, hemoptysis, or vitreous hemorrhage within 2 months<br>4) Major surgery within 6 weeks<br>5) History of intracranial bleeding, intracranial neoplasm, intracranial arteriovenous malformation, or intracranial aneurysm<br>6) Anemia (hemoglobin < 10 g/dL) or platelet count of less than 100,000/mm^3 at the time of screening<br>7) Concomitant treatment with oral anticoagulant agent (vitamin-K antagonists or novel oral anticoagulants such as dabigatran, rivaroxaban, apixaban, or edoxaban<br>8) Daily treatment with non-steroidal anti-inflammatory drugs (NSAIDs) or cyclooxygenase-2 inhibitors<br>9) Malignancy or life expectancy of less than one year<br>10) Moderate or severe hepatic dysfunction (Child Pugh B or C)<br>11) Symptomatic patients with sinus bradycardia (sick sinus syndrome) or atrioventricular (AV) block (AV block grade II or III, bradycardia-induced syncope; except for patients implanted with permanent pacemaker)<br>12) Symptomatic patients with chronic obstructive pulmonary disease (Medical research council grade ≥3)<br>13) Intolerance of or allergy to aspirin, ticagrelor or clopidogrel<br>14) Subjects who are under renal replacement therapy due to end-stage renal disease or who have history of kidney transplantation<br>15) Galactose intolerance, lactase insufficiency or glucose-galactose malabsorption<br>16) Subjects who are actively participating in another clinical trial within 3 months of randomization (except for observational study)<br>17) Pregnant and/or lactating women

<table>
<tr><td></td><td>18) Subjects considered unsuitable for this study by the investigator</td></tr>
<tr><td>Study Design</td><td>

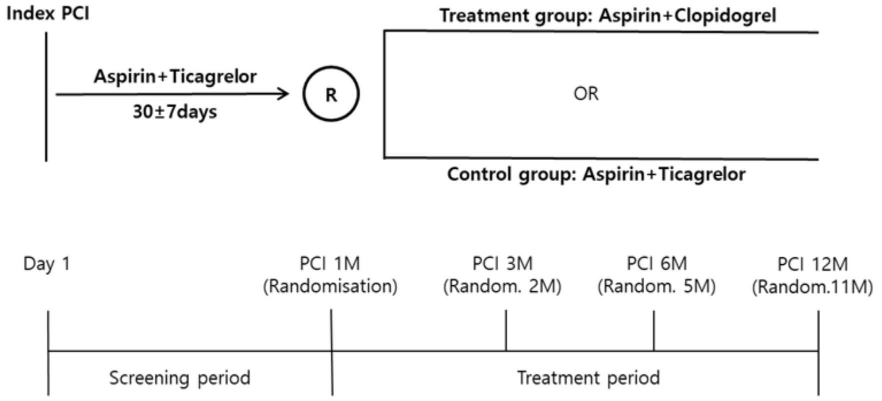

Screening period
To conduct screening AMI patients based on the inclusion/exclusion criteria who (1) have been treated with ticagrelor+aspirin for at least 30±7 days after an index PCI, (2) received full explanation of the study details, (3) given written consent.

To randomize eligible subjects within 30±7 days after AMI undergoin g PCI with newer generation DES, and receiving aspirin and ticagre lor to the treatment and control groups in a 1:1 ratio.
Treatment period
Enrolled patients receive clopidogrel 75mg + aspirin 100 mg (treatment group) or ticagrelor 90mg bid +aspirin 100mg treatment (control group) for 11 months (post-AMI 1 month to 12 months) and evaluation safety and efficacy by conducting physical examination, checking vital sign, and collecting adverse events at post-PCI 3M, 6M, 12M visits.

Labboratory and imaging tests, which undergo according to the medical judgment of each investigator during the study period, are collected by reviewing medical records or EMR.

</td></tr>
<tr><td>Evaluation Standard</td><td><u>Efficacy Test Variables</u>
1) Primary Endpoint (net clinical benefit)
Composite endpoint of MACCE (CV death, MI, or stroke) + BARC bleeding (type 2, 3 or 5) between 1 and 12 months after AMI</td></tr>
</table>

	2) Main Secondary Endpoints<br>① BARC bleeding (type 2, 3, or 5) between 1 and 12 months after AMI<br>② Composite endpoint of MACCE (CV death, MI, or stroke) + BARC bleeding (type 3, 5) between 1 and 12 months after AMI<br>③ Composite endpoint of MACCE (CV death, MI, or stroke) between 1 and 12 months after AMI<br>3) Other Secondary Endpoints<br>① All-cause death between 1 and 12 months after AMI<br>② CV death between 1 and 12 months after AMI<br>③ Recurrent MI between 1 and 12 months after AMI<br>④ Stroke between 1 and 12 months after AMI<br>⑤ Ischemia Driven Revascularization including PCI or CABG between 1 and 12 months after AMI<br>⑥ Stent thrombosis (definite or probable) between 1 and 12 months after AMI<br><br>4) Exploratory Test Items<br>① Lab test<br>② Echocardiogram<br>③ ECG<br>④ Genetic test<br><br>**<u>Safety Test Variables</u>**<br>1) Vital sign<br>2) Physical examination<br>Adverse event
**Statistical Analysis**	**<u>Efficacy Test Variable Analysis</u>**<br>1. Primary endpoint analysisEfficacy Test<br>• The non-inferiority test between 1 and 12 months after AMI will be based on the Kaplan-Meier estimates. A 95% two-sided confidence interval will be computed for the difference event rate (clopidogrel + aspirin) – event rate (ticagrelor + aspirin). The clopidogrel group will be judged as non-inferior to the ticagrelor if the upper confidence limit is less than the

	predetermined non-inferiority margin of 3% (absolute risk difference).<br>• The hypothesis of non-inferiority test will be based on the difference of proportions. Let r_T denote the true event proportion in the test arm (clopidogrel + aspirin) between 1 and 12 months, and r_C denote the true event proportion in the control arm (ticagrelor + aspirin) between 1 and 12 months. The hypotheses are<br>H_0: r_T - $r_C \geq \Delta$<br>H_A: r_T - $r_C < \Delta$<br>The$\Delta$is the non-inferiority margin, and is taken to be 0.03. The test will be performed as a one-sided test at alpha=0.05.<br>The null hypothesis shall be rejected at alpha=0.05 if the one-sided p-value is less than 0.05. When this occurs, the upper limit of the two-sided 95% confidence interval will be less than 3%.<br>• If the non-inferiority analysis passed the acceptance criterion, a superiority analysis will be performed. Statistical superiority is achieved when the upper limit of the two-sided 95% confidence interval of the risk difference is less than 0%. The type I error for this analysis is protected by the non-inferiority analysis, and no alpha adjustment would be appropriate<br>• Subgroup analyses will be performed by the primary endpoint categorized by type of AMI (STEMI vs NSTEMI), Subgroup analyses will be performed by the primary endpoint categorized by Type of AMI (STEMI vs NTEMI), Gender, Age (≥75 vs <75), Diabetes, LVEF (≥40% vs <40%), eGFR (≥60 vs <60), type of implanted stents (Xience vs Resolute vs Synergy stents), Bleeding risk according to the ARC criteria (high vs low bleeding risk), CYP2C19 loss-of-function carrier status (carrier vs non-carrier).<br>• The primary analysis population for primary and secondary endpoints will be the Intention-to-Treat (ITT) population. The primary endpoint analysis will also be performed on the Per Protocol (PP) population as subsequent analysis.<br>• A primary endpoint analysis stratified by the institutions as a sensitivity analysis. Strata will be divided by the accrual number of institution based

	on quartiles.<br>Implement noninferiority validation based on the tolerance limit after collecting cumulative occurrence rate of MACCE (CV death, MI, stroke) + BARC bleeding (type 2, 3, or 5) post 1M-1Y PCI and checking 95% confidence interval of [Ticagrelor occurrence rate – Clopidogrel occurrence rate]. If the upper value of the 95% confidence interval is less than 3% of the noninferiority tolerance limit, Clopidogrel is perceived noninferior to Tricagrelor. Present the cumulative limit method, Kaplan-Meier curve and conduct log-rank test to check the difference between two groups.<br><br>2. Main Secondary Endpoint Analyses<br>• The secondary endpoints will be composed of two families. The first family consists of the composite endpoint of MACCE (CV death, MI, stroke) plus BARC bleeding (type 2, 3, or 5). The second family will consist of MACCE plus BARC bleeding (type3, or 5), MACCE, and BARC bleeding (type 2, 3, or 5). The endpoints from the second family will be tested hierarchically, thereby maintaining the study-wise alpha level. These secondary endpoints will only be tested if both the primary composite endpoint and BARC bleeding are significant at non-inferiority analysis, and superiority analysis. Composite endpoint of MACCE plus BARC bleeding (type 3, or 5) will be tested first, and only if this is significant, the composite endpoint of MACCE only will be tested afterwards. BARC bleeding (type 2, 3, or 5) will be tested only if both of the above endpoints are tested significant.<br><br>3. Exploratory Test Variable<br>For continuous data, present technical statistics (mean, standard deviation, median, min. and max. value) on the changes between base & each visits. Based on whether the data follow normal distribution, comparatively verify between the two groups using the independent t-test or Wilcoxon’s rank sum test. Also, for comparison within each group, analyze using the paired t-test or Wilcoxon’s signed rank test. For c ategorical data, present technical statistics (frequency, percentage) an d comparatively verify between groups using the chi-square test or Fisher’s exact test.<br><br>4. Additional analysis should be run including all occurred events if the drug is given continuously.

	**<u>Safety Test Variable Analysis</u>**<br>1. Adverse Event<br>Should be conducted for all adverse events occurred during clinical test. Summarize adverse event occurrence rate, occurrence rate of specific adverse event causing discontinuation of drugs or loos to follow-up and occurrence rate of critical adverse event. Adverse event occurrence rate includes all adverse event occurrence rate and occurrence rate of adverse event related to the clinical test drug.<br>2. Vital Sign, Physical Examination<br>For continuous data, present technical statistics (mean, standard deviation, median, min. and max. value) on the changes between base & each visits. Based on whether the data follow normal distribution, comparatively verify between the two groups using the independent t-test or Wilcoxon's rank sum test. Also, for comparison within each group, analyze using the paired t-test or Wilcoxon's signed rank test. For categorical data, present technical statistics (frequency, percentage) and comparatively verify between groups using the chi-square test or Fisher's exact test.<br>**<u>(interim analysis is not performed.)</u>**<br><br>**<u>Analysis Population</u>**<br>1. The Intent to Treat (ITT) Population<br>The ITT population is defined as all randomized patients at 1 month after AMI, regardless of their adherence with the entry criteria, regardless of treatment they actually received, and regardless of subsequent withdrawal from treatment or deviation from the protocol[19]. Only some specific reasons that might cause an exclusion of a patient from the ITT population:<br><br>● No treatment was applied at all<br>● No data are available after randomization<br><br>2. The Per Protocol (PP) Population<br>The PP population is the subset of ITT population consisting of all patients who receive and retain the treatment during 12 months after PCI[19]. Some specific reasons that might cause an exclusion of a patient from the PP population:<br>● Violation of entry criteria including inclusion and exclusion criteria<br>● Withdrawal of consent

	● Concomitant treatment of oral anticoagulant agent (vitamin-K antagonists or novel oral anticoagulants such as dabigatran, rivaroxaban, apixaban, or edoxaban) during the study period<br>● Poor compliance<br>- Conversion from ticagrelor + aspirin to clopidogrel + aspirin during RCT procedure and vice versa<br>- Discontinuation of test or control drugs for 7 days or longer<br>3) * In the cases of withdrawal of consent, concomitant treatment of or al anticoagulation agent and poor compliance, their data will be used fo r statistical analyses until such events occur.

134

DEFINITION

A	Peak late diastolic velocity
AE	Adverse Event
ADP	Adenosine diphosphate
AST	Aspartate aminotransferase
ALT	Alanine aminotransferase
BARC	Bleeding Academic Research Consortium
CABG	Coronary Artery Bypass Graft surgery
CRO	Contract Research Organization
DES	Drug Eluting Stent
DT	Deceleration time
E	Peak early diastolic velocity
E'	Early diastolic velocity of mitral annulus
EF	Ejection fraction
GCP	Good Clinical Practice
Hb	Hemoglobin
IRB	Institutional Review Board
ITT	Intent-To-Treat
IWRS	Interactive web-based response system
LVEDV	Left Ventricle end-diastolic volume
LVESV	Left Ventricle end-systolic volume
MACCE	Major Adverse Cardiac and Cerebrovascular event
MI	Myocardial Infarction
NSTEMI	Non-ST Elevation Myocardial Infarction
PCI	Percutaneous Coronary Intervention
PLATO	A study of PLATelet inhibition and Patient Outcomes
PP	Per Protocol
RVSP	Right Ventricular systolic pressure
SAE	Serious Adverse event
STEMI	ST Elevation Myocardial Infarction
TRITON-TIMI	TRial to Assess Improvement in Therapeutic Outcomes by Optimizing Platelet InhibitioN with Prasugrel–Thrombolysis In Myocardial Infarction

TIME TABLE

Schedule of Measurements		Screening	Baseline	Treatment		
		V1	V2	V3	V4	V5
		-30D ~ -1D (PCI)	1D* (PCI ±30 days)	2M† (PCI ± 3M)	5M† (PCI ± 6M)	11M‡ (PCI ± 12M)
Informed Consent		●				
Demographics		●				
Physical Examination[1)]		●	●	●	●	●
Medical History		●				
Current Medication		●				
Dyspnea Evaluation		●	●	●	●	●
Subject Suitability Test		●	●			
Pregnancy Test[2)]		●				
Randomization			●			
Efficacy Test[3)]			●	●	●	●
Exploratory Test[4)]		●	●	●	●	●
Safety Test	Vital Sign	●	●	●	●	●
	Physical Examination	●	●	●	●	●
	Adverse Event Test		●	●	●	●
Investigational Product Prescription			●	●	●	
Investigational Product Adherence Assessment				●	●	●
Concomitant Medication Change Test			●	●	●	●

***: Post** PCI 30 days ±7 days
†: ±14 days permitted from identified date. Call possible if visits are not feasible. Identify in Case Report Form
‡ :– 14 days ~ + 30 days permitted from identified date. Call possible if visits are not feasible. Identify in Case Report Form.

1) Measure weight at each visit
2) Pregnancy Test: Conduct urine β-HCG test among fertile women who have not identified menopause (no period for 12M or longer)
3) Efficacy Test: Stroke, BARC bleeding (type 2, 3, or 5), Cardiac death, Death from any cause, Death from vascular cause, Acute MI, Stent thrombosis, Ischemia Driven Revascularization
4) Exploratory Endpoint: Lab Test, Cardiac Echo, ECG, Genetic Test (Optional)

i. Lab Test

① Blood Test: WBC, Neutrophil, Lymphocytes, Hemoglobin, Platelet, ESR

② Blood Coagulation Test: INR, Fibrinogen

③ Blood Chemical Test: Glucose, Creatinine, AST, ALT, Total bilirubin, Alkaline phosphatase, γ-GTP, Calcium, Phosphorus, LDH, CPK, Uric acid, Total cholesterol, Triglyceride, HDL-cholesterol, LDL-cholesterol, hsCRP

④ Glycosylated hemoglobin

⑤ Platelet Function Test: VerifyNow, PFA-100/200

⑥ Myocardial Damage Index Test

⑦ Thyroid Function Test

⑧ Kidney Function Test: Cockcroft-Gault eCCr, MDRD eGFR

- Cockcroft-Gault eCCr (ml/min) = (140-age) * (Weight in kg) / (72 * SCr) * (0.85 for women)
- MDRD eGFR(mL/min/1.73m^2) = 186 * (SCr)$^{-1.154}$ * (Age)$^{-0.203}$ * 0.742(for women)

ii. Cardiac Echo

iii. ECG

5) Institution conducting genetic tests for analysis should receive subject consent form (Optional).

Title and Phase of Clinical Trial

A Prospective, multicenter, Randomized, Open-label Trial to Compare Efficacy and Safety of Clopidogrel vs Ticagrelor in stabilized Patients with Acute Myocardial Infarction (AMI) after Percutaneous Coronary Intervention (PCI); **T**ic**A**grelor versus **CLO**pidogrel in Stabilized patients with **A**cute **M**yocardial **I**nfarction: **TALOS-AMI**

Phase 4

1. Study Institution

<Appendix 1> Reference

2. Principal investigator, Sub-investigator and Clinical Research Coordinator

2.1. Principal investigator

Name	Institution	Specialty (Division)	Position
Kiyuk Chang*	Seoul St. Mary's Hospital, The Catholic University of Korea	Cardiology	Professor

*Coordinating Investigator (CI) of Clinical Trial

2.2. Sub-investigator and Clinical Research Coordinator

<Appendix 1> Reference

3. Sponsor

Seoul St. Mary's Hospital, 06591, 222 Banpo-daero, Seocho-gu, Seoul

4. Background and Objective

4.1. Objective

To investigate the efficacy and safety of switching from ticagrelor to clopidogrel in stabilized patients with AMI with no adverse events during the first month after an index PCI

4.2. Background

In AMI, adequate platelet inhibition is essential to reduce the risk of recurrent thrombotic events. For this reason, dual antiplatelet therapy (DAPT) with aspirin and a P2Y12 inhibitor has become the current mainstay of pharmacological treatment in AMI patients managed with PCI. Although, clopidogrel has an indication for use in AMI[1], potent P2Y12 inhibitors, ticagrelor and prasugrel, compared with clopidogrel have shown significantly improved clinical outcomes in terms of reducing recurrent ischemic events in large randomized trials[2,3]. Thus, current guidelines strongly recommended potent P2Y12 inhibitors for 1 year in AMI patients undergoing PCI[1].

However, along with strong anti-platelet efficacy, a higher risk for bleeding was observed for potent P2Y12 inhibitors compared with clopidogrel in these randomized trials. Intriguingly, benefit due to reduction of ischemic events and harm due to bleeding events predominates at different time points during potent P2Y12 inhibitors treatment[4]. Although the ischemic benefit was consistent throughout the first year after the index event, the benefit of ticagrelor and prasugrel over clopidogrel for reducing thrombotic risk was prominent in the early period after acute coronary syndrome (ACS) when the risk of ischemic complications was highest. In the primary PCI cohort of the PLATO (Platelet Inhibition and Patient Outcomes) trial, ticagrelor showed larger risk reduction for stent thrombosis (ST) during the first 30days of treatment compared with clopidogrel but the difference decreased over time[5]. Similarly, in the TRITON-TIMI (Therapeutic Outcomes by Optimising Platelet InhibitioN with Prasugrel-Thrombolysis In Myocardial Infarction) 38 trial, prasugrel led to a 25% reduction in MI during the first month[6]. On the other hands, the opposite was true for bleeding. Landmark analyses of these two randomized trials revealed that the bleeding risk was similar in the early period of treatment, but there was a larger difference during the chronic period of treatment between potent P2Y12 inhibitors and clopidogrel. Actually most bleeding events predominantly occurred during the maintenance period of treatment[7,8]. As a consequence, to optimize net clinical benefit between early ischemic benefit and late bleeding risks in AMI patients, many physicians have focused on the novel therapeutic strategy of stepwise de-escalation using potent P2Y12 inhibitors only in the acute phase of treatment (during the first 30 days) and using the less potent clopidogrel during the chronic phase of treatment (after the first 30 days).

Despite of the evidence for the consistent efficacy and safety of potent P2Y12 inhibitors with long-term treatment, de-escalation after ACS is quite common in clinical practice[9-12]. Data have shown that the prevalence of de-escalation during hospitalization ranges from 5% to 14%[10,11] and after discharge ranges from 15% to 28%[12]. However, at present, data from large-scale clinical studies on the topic of de-escalating antiplatelet strategy are very limited and the results of small studies are conflicting[9,13]. Recently, some randomized trials of de-escalation enrolling ACS patients have been reported[13,14]. The randomized, open-label, single-center TOPIC trial (Timing of Optimal

Platelet Inhibition After Acute Coronary Syndrome) showed that in patients who have been event free for the first month after an ACS on a combination of aspirin plus a potent P2Y12 inhibitor (ticagrelor or prasugrel), de-escalation to aspirin plus clopidogrel strategy was associated with reduction of bleeding complications without increase in ischemic events[13]. Although this study did not show any differences in ischemic events between groups, play of chance cannot be ruled out given the limited sample size of the trial[15]. The open-label, multicenter TROPICAL-ACS trial (Testing Responsiveness to Platelet Inhibition Chronic Antiplatelet Treatment for ACS) randomized patients with ACS undergoing PCI to either standard treatment with prasugrel for 12 months or a de-escalation regimen (1 week of prasugrel followed by 1 week of clopidogrel and platelet function testing [PFT]–guided maintenance therapy with clopidogrel or prasugrel from day 14 after hospital discharge)[14]. The trial showed that a strategy of PFT-guided de-escalation of antiplatelet treatment was noninferior to standard treatment with prasugrel at 1 year in terms of net clinical benefit. The PFT-guided de-escalation strategy did not show any increase in ischemic events, although there was not a statistically significant reduction in bleeding. However, some experts expressed concerns about a lack of power due to the low number of endpoint events[16]. Furthermore, the routine use of PFT in ACS patients undergoing PCI is limited because it is not widely available in real world clinical practice. And, although prasugrel and ticagrelor have similar levels of platelet inhibition, it might be argued that the study findings cannot be applied to ticagrelor because its pleiotropic effects have been advocated to contribute to its overall benefits[17]. In addition, there are no studies for the de-escalation of antiplatelet treatment enrolling only AMI patients treated by PCI with newer generation DES. In PLATO study, only 60% of population were scheduled for PCI and the patients who underwent PCI received older generation DES.

Therefore, we sought to investigate the efficacy and safety of switching from ticagrelor to clopidogrel in AMI patients with no adverse event during the first month after index PCI with second generation DES.

5. Study Drug

5.1. Test Drug

Test Product	Pregrel
Component	Clopidogrel resinate 150mg (75mg as)
Description and dose form	Pinkish film coated circular pill
Storage Method	Air tight container, room temperature (1~30℃)
Efficacy and Effect	Improvement of clinical outcomes (cardiovascular death, myocardial infarction, stroke, refractory ischemia) in patients with acute coronary syndrome patients who are medically treated or have received PCI or CABG

5.2. Comparator

Test Product	Brilinta
Component	Ticagrelor 90mg
Description and dose form	Yellowish film coated pill with convex sides
Storage Method	Air tight container, room temperature (1~30℃)
Efficacy and Effect	Reduction of thromboembolic cardiovascular event (cardiovascular death, myocardial infarction, or stroke) in patients with acute coronary syndrome who are planned to receive pharmacotherapy, PCI or CABG in addition to aspirin.

6. Study Disease

Acute Myocardial Infarction

<3rd Universal Definition of Myocardial Infarction>[18]

**_Criteria for acute myocardial infarction**
The term acute myocardial infarction (MI) should be used when there is evidence of myocardial necrosis in a clinical setting consistent with acute myocardial ischemia.<br>Under these conditions any one of the following criteria meets the diagnosis for MI:<br><br>• Detection of a rise and/or fall of cardiac biomarker values [preferably cardiac troponin (cTn)] with at least one value above the 99th percentile upper reference limit (URL) and with at least one of the following:<br>■ Symptoms of ischemia.<br>■ New or presumed new significant ST-segment–T wave (ST–T) changes or new left bundle branch block (LBBB).<br>■ Development of pathological Q waves in the ECG.<br>■ Imaging evidence of new loss of viable myocardium or new regional wall motion abnormality.<br>■ Identification of an intracoronary thrombus by angiography or autopsy.<br>• Cardiac death with symptoms suggestive of myocardial ischemia and presumed new ischemic ECG changes or new LBBB, but death occurred before cardiac biomarkers were obtained, or before cardiac biomarker values would be increased.<br>• Percutaneous coronary intervention (PCI) related MI is arbitrarily defined by elevation of cTn values (_>5 x 99th percentile URL) in patients with normal baseline values (≤_99th percentile URL) or a rise of cTn values _>20% if the baseline values are elevated and are stable or falling. In addition, either (i) symptoms suggestive of myocardial ischemia or (ii) new ischemic ECG changes or (iii) angiographic findings consistent with a procedural complication or (iv) imaging demonstration of new loss of viable myocardium or new regional wall motion abnormality are required.<br>• Stent thrombosis associated with MI when detected by coronary angiography or autopsy in the setting of myocardial ischemia and with a rise and/or fall of cardiac biomarker values with at least one value above the 99th percentile URL.<br>• Coronary artery bypass grafting (CABG) related MI is arbitrarily defined by elevation of cardiac biomarker values (>10 x 99th percentile URL) in patients with normal baseline

cTn values (≤99th percentile URL). In addition, either (i) new pathological Q waves or new LBBB, or (ii) angiographic documented new graft or new native coronary artery occlusion, or (iii) imaging evidence of new loss of viable myocardium or new regional wall motion abnormality.

7. Inclusion/Exclusion Criteria & Study Population

7.1. Subject Inclusion Criteria

Subject should meet all of the following criteria.

1) Age ≥ 18 years
2) Patients with AMI (STEMI or NSTEMI) who are administered aspirin and ticagrelor for 30 days after successful PCI with newer-generation drug eluting stents (DES)
   *Definition of AMI follows the 3rd Universal Definition of MI.
3) Female patients with childbearing potential who agree to mandatory pregnancy test and have committed to using adequate contraception
4) Subjects who agree to the study protocol and the schedule of clinical follow-up, and provides informed, written consent, as approved by the appropriate IRB of the respective institution

7.2. Subject Exclusion Criteria

Subject should be excluded if they apply to any of the following criteria.

1) Cardiogenic shock
2) Active internal bleeding, bleeding diathesis, or coagulopathy
3) Gastrointestinal bleeding or genitourinary bleeding, hemoptysis, or vitreous hemorrhage within 2 months
4) Major surgery within 6 weeks
5) History of intracranial bleeding, intracranial neoplasm, intracranial arteriovenous malformation, or intracranial aneurysm
6) Anemia (hemoglobin < 10 g/dL) or platelet count of less than 100,000/mm^3 at the time of screening
7) Concomitant treatment with oral anticoagulant agent (vitamin-K antagonists or novel oral anticoagulants such as dabigatran, rivaroxaban, apixaban, or edoxaban)
8) Daily treatment with non-steroidal anti-inflammatory drugs (NSAIDs) or cyclooxygenase-2 inhibitors
9) Malignancy or life expectancy of less than one year
10) Moderate or severe hepatic dysfunction (Child Pugh B or C)

11) Symptomatic patients with sinus bradycardia (sick sinus syndrome) or atrioventricular (AV) block (AV block grade II or III, bradycardia-induced syncope; except for patients implanted with permanent pacemaker)
12) Symptomatic patients with chronic obstructive pulmonary disease (Medical research council grade ≥3)
13) Intolerance of or allergy to aspirin, ticagrelor or clopidogrel
14) Subjects who are under renal replacement therapy due to end-stage renal disease or who have history of kidney transplantation
15) Galactose intolerance, lactase insufficiency or glucose-galactose malabsorption
16) Subjects who are actively participating in another clinical trial with 3 months of randomization (except for observational study)
17) Pregnant and/or lactating women
18) Subjects considered unsuitable for this study by the investigator

7.3. Study Population

7.3.1. Sample Size

	Test	Control	Total Sample Size
No. of efficacy case	1165	1165	2330
Including follow-up loss rate (10%)	1295	1295	2590

7.3.2. Sample Size Estimation

The present study is designed to show noninferiority of the treatment group with aspirin plus clopidogrel versus the control group with aspirin plus ticagrelor. Sample size is based on the combined occurrence rate of ischemic and bleeding events between 1 and 12 months after AMI. According to the PLATO investigators, the event rate of primary efficacy endpoint including CV death, MI or stroke was 5.28% in the ticagrelor group and 6.60% in the clopidogrel group between 1and 12 months after the index event[2]. In the meantime, since there were no reported data on the bleeding event rate associated with ticagrelor from 1 to 12months after AMI, especially BARC bleeding rate at the time of the present study design, we assumed the event rate of BARC 2, 3 and 5 bleeding from the event rates of non-CABG related PLATO major or minor bleeding during a year of ticagrelor therapy (8.7%) and non-CABG related major bleeding of first 30 days (2.47%) and after 30 days (2.17%) in the PLATO trial. For the event rate of BARC 2, 3 and 5 bleeding associated with clopidogrel from 1 to 12 months after AMI, the event rate was assumed from the event rates of non-CABG related PLATO major or minor bleeding during a year of clopidogrel therapy (7.0%) and non-

CABG related major bleeding of first 30 days (2.21%) and after 30 days (1.65%) in the clopidogrel group of the PLATO trial[7]. We applied mathematical formula for the estimation of the event rate of BARC 2, 3, 5 bleeding from the event rates of non-CABG related PLATO major or minor bleeding: "In the ticagrelor group

non-CABG major bleeding first 30 days : non-CABG major bleeding after 30days = 2.47 : 2.17

non-CABG total bleeding first 30 days : non-CABG total bleeding after 30days = (8.7- χ) : χ

$$2.47:2.17 = (8.7-\chi) : \chi$$

$$\chi = 4.07\%$$

In the clopidogrel group

non-CABG major bleeding first 30 dasy : non-CABG major bleeding after 30 days = 2.21:1.65

non-CABG total bleeding first 30 days : non-CABG total bleeding after 30 days = (7.0- χ) : χ

$$2.21:1.65 = (7.0-\chi) : \chi$$

$$\chi = 2.99\%$$"

After applying mathematical formula, the estimated BARC 2, 3 and 5 bleeding would be 4.07% in the ticagrelor group and 2.99% in the clopidogrel group. Thus, the expected event rate of the primary endpoint from 1 to12 months after index PCI was 9.35% (ischemic event of 5.28% + bleeding event of 4.07%) in the ticagrelor group and 9.59% (ischemic event of 6.6% + bleeding event of 2.99%) in the clopidogrel group. We chose the noninferiority margin in accordance with clinical judgment and other relevant studies with a noninferiority design at the present study design. The noninferiority margin of two contemporary trials of antiplatelet treatment after PCI that were available up to that time was equivalent to a 40% increase in the expected event rate[18, 19]. The steering committee decided that the noninferiority margin in our study should be less than a 40% increase compared to the expected event rate of the control group. After considering clinically acceptable relevance and the feasibility of study recruitment, we finally selected the noninferiority margin of 3.0%, which was equivalent to a 32% increase in the expected event rate. Sample size calculations (PASS 13, NCSS, LLC, Kaysville, Utah, USA) were performed based on one-sided α of 0.05 and a power of 80 %. To achieve these goals, a total of 2,230 patients were needed. After considering a follow-up loss rate of 10%, a total of 2,590 (1,295 patients in each group) patients were required.

8. Study Duration

IRB approval to Dec. 31st, 2020

9. Study Method

9.1. Study Process

Phase IV

9.2. Study Design

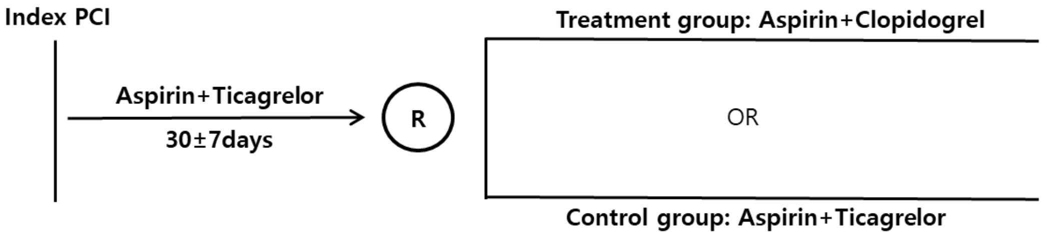

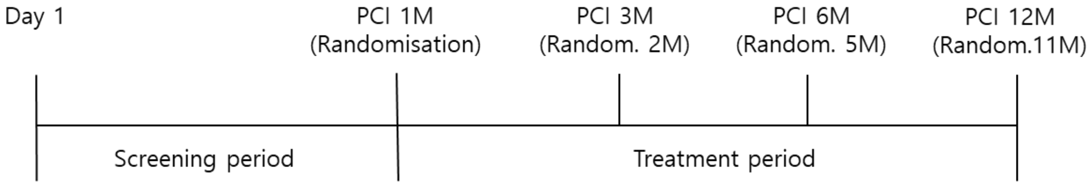

- **Screening period**

  To conduct screening AMI patients based on the inclusion/exclusion criteria who (1) have been treated with ticagrelor + aspirin for at least 30±7 days after an index PCI, (2) received full explanation of the study details, (3) given written consent.

  To randomize eligible subjects within 30±7 days after AMI undergoing PCI with newer generation DES, and receiving aspirin and ticagrelor to the treatment and control groups in a 1:1 ratio.

- **Treatment period**

  Enrolled patients receive clopidogrel 75mg + aspirin 100 mg (treatment group) or ticagrelor 90mg bid +aspirin 100mg treatment (control group) for 11 months (post-AMI 1 month to 12 months) and evaluation safety and efficacy by conducting physical examination, checking vital sign, and collecting adverse events at post-PCI 3M, 6M, 12M visits.

  Laboratory and imaging tests, which undergo according to the medical judgment of each investigator during the study period, are collected by reviewing medical records or EMR.

9.3. Randomization

9.3.1. Subject Assignment and Randomization

Randomization will be performed to ensure the scientific validity of the clinical test. This will maximize the comparability of the test and control group and eliminate the subjectivity of the researchers in subject group assignment. Before PCI, a 250-325mg loading dose of aspirin is given to patients who are naïve to treatment and all patients receive a loading dose of ticagrelor 180mg. Discharge medication consists of aspirin 100mg once and ticagrelor 90mg twice per day. All patients receive treatment with aspirin plus ticagrelor for 1 month after the index PCI (screening period). At 30 ± 7days after index PCI, eligible patients were randomly assigned either to the 1) aspirin 100 mg plus clopidogrel 75mg daily (treatment group) or 2) aspirin 100 mg plus ticagrelor 90mg twice daily (control group) in a 1:1 ratio. Randomization will occur centrally. To randomize a patient, the investigative site will enter the subject into the designated electronic system and obtain the treatment assignment (clopidogrel + aspirin or ticagrelor + aspirin) in a 1:1 ratio. At 1 month visit after AMI, eligible subjects were assigned to each treatment group following an access to the interactive web-based response system (IWRS, Medical Excellence Inc., Seoul, Korea) by the investigator or designee. Randomization sequence was created by an independent statistician using SAS 9.3 (SAS Institute Inc. Cary, NC, USA) statistical software and was stratified by study center and type of AMI (STEMI or NSTEMI) and with a 1:1 allocation using hidden random block size.

9.3.2. Management and collection of Randomization

IWRS system is run by a 3rd party and the investigator receives subjects' consent, collects information required to select the subjects based on the inclusion/exclusion criteria and records test opinions during the screening phase. Subjects receive the screening number in order at this time. Final selection is conducted after reviewing the suitability of the subject and after that, subjects are assigned and given assignment numbers based on the randomization method. Consequently, subjects are assigned groups with their assignment number, based on the randomization table run by a 3rd party.

9.4. Dosage and Method

1) Test (Pregrel): 75mg oral administration, once a day
2) Control (Brilinta): 1 tablet (90mg) oral administration, twice a day

9.5. Switching protocol (ticagrelor to clopidogrel)

In the control treatment group, when switching from ticagrelor to clopidogrel, patients take a 75mg clopidogrel without loading dose at the time of the next scheduled dose after the final dose of ticagrelor (eg, ≈12 hours from last dose of ticagrelor). The steering committee decided this switching strategy of no loading dose based on the concept that our study population would be at

stable status at the time point of randomization (30 days after index PCI). The data safety and monitoring board (DSMB) approved this switching strategy on the condition that initial 100 enrolled patients in the treatment group should be monitored daily during first 7days for the occurrence of adverse clinical events by telephone interviews. Thereafter, DSMB reviewed the clinical data of the initial 100 patients in the treatment group and recommended continuation of the study according to the original protocol. After randomization, patients continue the same medication for 11 months according to their group allocation (treatment period, Figure 1). Patients are evaluated at 3 (2 months after randomization), 6 (5 months after randomization), and 12 (11months after randomization) months after index PCI and monitored for the occurrence of the clinical events.

9.6. Combination Treatment and Cautions

All medication at the time of enrollment and during the trial, other than the investigational drugs, should be considered as a combination therapy and must be recorded in the case record and medical record (general name, route of administration, administrating start and modification date, daily dose, etc). Administration of concomitant medications should be minimized during the clinical trial and changes to concomitant medication should be minimized except for essential drugs. The administration of drugs other than contraindicated medication is permitted.

Drugs prohibited during the clinical trial include:

1) Anticoagulants: Vitamin K antagonist, Direct thrombin inhibitor, factor X inhibitor, heparin (except for temporary use in PCI), low molecular-weighted heparin

2) Antithrombotic agent: Prasugrel, ticlopidine, beraprost, cilostazol, dipyridamole, Limaprost, α-cyclodextrin clathrate, Sarpogrelate, glycoprotein IIb/IIIa inhibitors

3) Corticosteroids (except locally use): betamethasone, cortisone, dexamethasone, hydrocortisone, methylprednisolone, prednisolone, triamcinolone, etc

4) Digoxin: Ticagrelor is known to increase the drug concentration of digoxin moderately.

5) Drug interaction to CYP450

a) Potent inhibitor of CYP3A: Ketoconazole, itraconazole, voriconazole, telithromycin, clarithromycin [but not erythromycin or azithromycin], nefazodone, ritonavir, saquinavir, nelfinavir, indinavir, atazanavir, or over 1 liter daily of grapefruit juice may increase the drug concentration of

ticagrelor and should not be taken concomitantly.

b) CYP3A substrate or derivative: Simvastatin or lovastatin at a dose of 40 mg/day or more with ticagrelor is not allowed because it increases the drug concentration and there is a possibility of drug side effects of statin itself. There are no restrictions on other statin treatment. A potent inducer of CYP3A (eg, rifampin/rifampicin, rifabutin, phenytoin, carbamazepine, phenobarbital) should not be taken concomitantly.

6) Nonsteroidal anti-inflammatory drugs: diclofenac, ibuprofen, indomethacin, ketoprofen, meloxicam, naproxen, celecoxib, etc.

10. Time table, clinical and laboratory measurement

All process should follow the below time table. However, if the prescheduled visits are not kept under unavoidable circumstances, should record detailed reasons.

10.1. Time table

Schedule of Measurements	Screening	Baseline	Treatment		
	V1	V2	V3	V4	V5
	-30D ~ -1D (PCI)	1D* (PCI ±30 days)	2M† (PCI ± 3M)	5M† (PCI ± 6M)	11M‡ (PCI ± 12M)
Informed Consent	●				
Demographics	●				
Physical Examination[1)]	●	●	●	●	●
Medical History	●				
Current Medication	●				
Dyspnea Evaluation	●	●	●	●	●
Subject Suitability Test	●	●			
Pregnancy Test[2)]	●				
Randomization		●			
Efficacy Test[3)]		●	●	●	●
Exploratory Test[4)]	●	●	●	●	●

Safety Test	Vital Sign	●	●	●	●	●
	Physical Examination	●	●	●	●	●
	Adverse Event Test		●	●	●	●
Investigational Product Prescription			●	●	●	
Investigational Product Adherence Assessment				●	●	●
Concomitant Medication Change Test			●	●	●	●

***: Post** PCI 30 days ±7 days

†: ±14 days permitted from identified date. Call possible if visits are not feasible. Identify in Case Report Form

‡ :– 14 days ~ + 30 days permitted from identified date. Call possible if visits are not feasible. Identify in Case Report Form.

1) Measure weight at each visit
2) Pregnancy Test: Conduct urine β-HCG test among fertile women who have not identified menopause (no period for 12M or longer)
3) Efficacy Test: Stroke, BARC bleeding (type 2,3or 5), Cardiac death, Death from any cause, Death from vascular cause, Acute MI, Stent thrombosis, Ischemia Driven Revascularization
4) Exploratory Endpoint: Lab Test, Cardiac Echo, ECG, Genetic Test (Optional)
   (1) Lab Test
      ① Blood Test: WBC, Neutrophil, Lymphocytes, Hemoglobin, Platelet, ESR
      ② Blood Coagulation Test: INR, Fibrinogen
      ③ Blood Chemical Test: Glucose, Creatinine, AST, ALT, Total bilirubin, Alkaline phosphatase, γ-GTP, Calcium, Phosphorus, LDH, CPK, Uric acid, Total cholesterol, Triglyceride, HDL-cholesterol, LDL-cholesterol, hsCRP
      ④ Glycosylated hemoglobin
      ⑤ Platelet Function Test: VerifyNow, PFA-100/200
      ⑥ Myocardial Damage Index Test
      ⑦ Thyroid Function Test
      ⑧ Kidney Function Test: Cockcroft-Gault eCCr, MDRD eGFR
         - Cockcroft-Gault eCCr(ml/min) = (140-age) * (Weight in kg) / (72 * SCr) * (0.85 for women)
         - MDRD eGFR(mL/min/1.73m^2) = 186 * (SCr)$^{-1.154}$ * (Age)$^{-0.203}$ * 0.742(for women)
   (2) Cardiac Echo
   (3) ECG
5) Institution conducting genetic tests for analysis should receive subject consent form (Optional).

10.2. Clinical and laboratory measurement

10.2.1. Informed (written) Consent, Demographics & Physical Examination

Before enrollment, investigator should explain the objectives and details in-depth and receive written consent. After the informed consent is acquired, the investigator should record date of consent and demographics such as subject initials, gender and date of birth and also physical measurements (height, weight) in the case report form.

10.2.2. Changes in Current & Combined Medication, Medical history

During screening visit, investigator should review subjects' medical records and document past 1-year medical history. Also, review and record cardiovascular and diabetic medications past 60 days and at every visit onwards, investigate and record in the case report form if there are any changes in the recorded medications or there are any additional cardiovascular and diabetic medications.

10.2.3. Subject Suitability Test (based on inclusion/exclusion criteria)

Based on the consent, demographics, medical history, combined medication, physical examination and lab tests, evaluate and record if subjects are eligible using the inclusion/exclusion criteria.

10.2.3.1. Pregnancy Test

Pregnancy test should be performed during the screening visit (V1). Fertile women who have not identified as menopause (no period for 12M or longer) should be negative in urine HCG test. Also, they should agree to use medically acceptable methods of birth control during clinical test and follow-up observation period and be given training on these conditions.

10.2.3.2. Dyspnea Evaluation

Dyspnea evaluation should be performed during screening (V1) baseline (V2) visits. Should check the existence, intensity and causes of dyspnea, MMRC and Borg Scale. MMRC (Modified Medical Research Council Dyspnea Scale) is 0-4, higher in scale indicating greater difficulty of breathing. Borg Scale is 0-10, which indicates the awareness of fatigue and difficulty of breathing during exercise. (appendix 2, 3). (13,14) MMRC Dyspnea evaluation should be carried out at every visit.

10.2.4. Efficacy variable measurement

1) Primary Endpoint

Composite endpoint of MACCE (CV death, MI, or stroke) + BARC bleeding (type 2, 3 or 5) between 1 and 12 months after AMI

4) Main Secondary Endpoints

- ④ BARC bleeding (type 2, 3, or 5) between 1 and 12 months after AMI
- ⑤ Composite endpoint of MACCE (CV death, MI, or stroke) + BARC bleeding (type 3, 5) between 1 and 12 months after AMI
- ⑥ Composite endpoint of MACCE (CV death, MI, or stroke) between 1 and 12 months after AMI

5) Other Secondary Endpoints

- ⑦ All-cause death between 1 and 12 months after AMI

⑧ CV death between 1 and 12 months after AMI

⑨ Recurrent MI between 1 and 12 months after AMI

⑩ Stroke between 1 and 12 months after AMI

⑪ Ischemia Driven Revascularization including PCI or CABG between 1 and 12 months after AMI

⑫ Stent thrombosis (definite or probable) between 1 and 12 months after AMI

Check bleeding, Ischemia driven revascularization, Cardiac death, Death from any cause, Death from vascular cause, Acute MI, Stroke, Stent thrombosis according to the BARC definition 3M, 6M and 12M post PCI and record in the case report form.

MACCE is the combined rate of cardiac death, death from vascular cause, Acute MI, Stoke and primary efficacy endpoint is the combined bleeding rate based on the MACCE and BARC at 12M. This is derived through statistical analysis.

7.1.1.

Bleeding according to the BARC definition is as follows[(15)].

7.1.2.

Table 1 BARC Definition

BARC Definition		
Type 0		No bleeding
Type 1		Bleeding that is not actionable and does not cause the patient to seek unscheduled performance of studies, hospitalization, or treatment by a health care professional; may include episodes leading to self-discontinuation of medical therapy by the patient without consulting a health care professional
Type 2		Any overt, actionable sign of haemorrhage (e.g. more bleeding than would be expected for a clinical circumstance; including bleeding found by imaging alone) that does not fit the criteria for Types 3, 4, or 5, but does meet at least one of the following criteria:<br>(1) requiring non-surgical, medical intervention by a health care professional<br>(2) leading to hospitalization or increased level of care<br>(3) prompting evaluation.
Type 3	Type 3a	Overt bleeding plus hemoglobin drop of 3 to <5*g/dL (provided hemoglobin drop is related to bleed)<br>Any transfusion with overt bleeding

	Type 3b	Overt bleeding plus hemoglobin drop ≥ 5*g/dL (provided STEMI, NSTEMI drop is related to bleed)<br>Cardiac tamponade<br>Bleeding requiring surgical intervention for control (excluding dental/nasal/skin/hemorrhoid)<br>Bleeding requiring intravenous vasoactive agents
	Type 3c	Intracranial hemorrhage (does not include microbleeds or hemorrhagic transformation; does include intraspinal)<br>Subcategories; confirmed by autopsy or imaging or LP<br>Intra-ocular bleed compromising vision
Type 4		Coronary artery bypass graft-related bleeding<br>Perioperative intracranial bleeding within 48 hours<br>Reoperation after closure of sternotomy for the purpose of controlling bleeding<br>Transfusion of ≥5 U whole blood or packed red blood cells within a 48 hour period†<br>Chest tube output ≥2 L within a 24-hour period
Type 5	Type 5a	Probable fatal bleeding: no autopsy or imaging confirmation, but clinically suspicious
	Type 5b	Definite fatal bleeding: overt bleeding or autopsy or imaging confirmation

*****: Corrected for transfusion (1 U packed red blood cells or 1 U whole blood =1 g/dL hemoglobin)

† :Cell saver products are not counted.

7.1.3.

7.1.4.

Definite or probable according to the stent thrombosis definition us as follows[16].

Table 2 Stent Thrombosis Definition

Stent thrombosis	
Definite*	Angiographic confirmation of stent thrombosis†<br>The presence of a thrombus‡ that originates in the stent or in the segment 5 mm proximal or distal to the stent and presence of at least 1 of the following criteria within a 48-hour time window:<br>Acute onset of ischemic symptoms at rest<br>New ischemic ECG changes that suggest acute ischemia Typical rise and fall in cardiac biomarkers (refer to definition of spontaneous MI)

	Nonocclusive thrombus<br>Intracoronary thrombus is defined as a (spheric, ovoid, or irregular) noncalcified filling defect or lucency surrounded by contrast material (on 3 sides or within a coronary stenosis) seen in multiple projections, or persistence of contrast material within the lumen, or a visible embolization of intraluminal material downstream.<br><br>Occlusive thrombus<br>TIMI 0 or TIMI 1 intrastent or proximal to a stent up to the most adjacent proximal side branch or main branch (if originates from the side branch).<br><br>Pathological confirmation of stent thrombosis<br>Evidence of recent thrombus within the stent determined at autopsy or via examination of tissue retrieved following thrombectomy.
Probable	Clinical definition of probable stent thrombosis is considered to have occurred after intracoronary stenting in the following cases:<br>Any unexplained death within the first 30 days§<br>Irrespective of the time after the index procedure, any MI that is related to documented acute ischemia in the territory of the implanted stent without angiographic confirmation of stent thrombosis and in the absence of any other obvious cause

*Definite stent thrombosis is considered to have occurred by either angiographic or pathological confirmation.
†The incidental angiographic documentation of stent occlusion in the absence of clinical signs or symptoms is not considered a confirmed stent thrombosis (silent occlusion).
‡Intracoronary thrombus.
§For studies with ST-elevation MI population, one may consider the exclusion of unexplained death within 30 days as evidence of probable stent thrombosis.

7.1.5.

10.2.5. Safety monitoring

10.2.5.1. Vital Sign

At every visit, measure vital sign (blood pressure, pulse and respiratory rate measured sitting down for 5 min.)

10.2.5.2. Physical Examination

Physical examination should be conducted at every visit. Physical examination includes allergies, cardiovascular, lung/respiratory, gastrointestinal/liver, biliary, metabolic/endocrine, nephritic/urinary,

reproductive, musculoskeletal, skin/connective tissues, neurological, psychic and other physical organs. Results of clinical importance should be recorded in the comment box of the case report form. In case there are incidences of medical importance according to the adverse events definition after the test drug treatment, it should be recorded as adverse events in the case report form.

10.2.5.3. Adverse Event

The investigator should frequently train subjects to report proactively and check for adverse events through medical examinations during regular or additional visits. Reports of adverse event should include date of the adverse event began, date of the adverse event resolved, degree and result of the adverse event, actions taken related to the test drug, name of drug in question other than the test drug and treatment and contents of the adverse event. Major cardiovascular adverse events and bleeding adverse events should be recorded separately in the adverse event page in the case report form.

10.2.6. Exploratory Test Items

10.2.6.1. Lab Test

Based on the investigator’s medical judgment, following test results including the medical records should be recorded in the case report form. Most recent blood test, blood coagulation test, blood chemical test should be recorded.
Myocardial biomarker is collected at PCI admission during screening and if conducted at every visit, use the most recent result. Also collect thyroid function test if conducted.

Items of each test is as below.

① Blood Test: WBC, Neutrophil, Lymphocytes, Hemoglobin, Platelet, ESR
② Blood Coagulation Test: INR, Fibrinogen
③ Blood Chemical Test: Glucose, Creatinine, AST, ALT, Total bilirubin, Alkaline phosphatase, γ-GTP, Calcium, Phosphorus, LDH, CPK, Uric acid, Total cholesterol, Triglyceride, HDL-cholesterol, LDL-cholesterol, hsCRP
④ Glycosylated hemoglobin: HbA1c
⑤ Platelet Function Test: VerifyNow, PFA-100/200
⑥ Myocardial Damage Index Test: Maximum CK, Maximum CK-MB, Maximum Troponin I, Maximum Troponin T, NT-proBNP, BNP
⑦ Thyroid Function Test: T3, free T4, TSH
⑧ Kidney Function Test: Cockcroft-Gault eCCr, MDRD eGFR

- Cockcroft-Gault eCCr(ml/min) = (140-age) * (Weight in kg) / (72 * SCr) * (0.85 for women)
- MDRD eGFR(mL/min/1.73m^2) = 186 * (SCr)$^{-1.154}$ * (Age)$^{-0.203}$ * 0.742(for women)

10.2.6.2. Cardiac Echo

Collect below items if ECHO is conducted.

- EF (Ejection fraction)
- LVEDV (Left Ventricle end-diastolic volume)
- LVESV (Left Ventricle end-systolic volume)
- LVDd (diastolic left ventricular diameter)
- LVDs (systolic LV diameter)
- IVSd (diastolic interventricular septal wall thickness)
- PWTd (diastolic posterior wall thickness)
- RWT (Relative Wall Thickness)
- LVM (Left Ventricular Mass)
- S' (Systolic velocity of mitral annulus)
- GLS (Global Left ventricular Strain)
- E (Peak early diastolic velocity)
- A (Peak late diastolic velocity)
- DT (Deceleration time)
- E' (Early diastolic velocity of mitral annulus)
- RVSP (Right Ventricular systolic pressure)
- LA diameter(Left Atrial diameter)
- LA volume index
- peak TR regurgitation velocity
- Tei index(Myocardial Performance Index)

10.2.6.3. ECG

- Basic rhythm
- Ventricular rate
- PR interval
- QRS duration
- QT
- QTc
- QRS axis

10.2.6.4. Genetic Test

If a subject agrees to the genetic test, blood sample is collected once during the trial and store for future genetic analysis associated with pharmacogenetics of clopidogrel or ticagrelor (CYP2C19, CY P2B6, CYP3A4, CYP3A5, P2RY12, and ABCB1) and exploration related to occurrence of MI using single-bse extension methods. It should be conducted in the central lab and there could be additional tests under regulatory or medical perspective. Investigator should follow the lab manual for details of storage and transportation. Genetic tests is planned to proceed at "Catholic Cardiovascular Research Institute for Intractable Disease (CRID) of Seoul St. Mary's Hospital. 6-10mL of sample should be collected and mixed well in a Becton Dickinson (BD) vacutainer tube. This should be separated and kept in BD falcon tubes in -80°C freezer. Samples should be transferred from each site to Seoul St. Mary's Hospital (Central) every 6 months. Storage period is 5 years from the day of transport and afterwards, disposed. If the consent is withdrawn after providing the specimen, samples will be disposed immediately with the request of the subject even before the termination of trial. However, analysis conducted before the withdrawal will be used in the research and no additional data will be collected after the withdrawal.

10.3. Visit schedule and assessment

10.3.1. 1st Visit (Screening, -30D ~ -1D)

1) Subject written consent
2) Demographic/Physical examination
3) Medical history
4) Current medication
5) Dyspnea evaluation
6) Pregnancy test
7) Vital sign
8) Physical examination
9) Subject suitability test
10) Exploratory test (Optional): Lab test, Cardiac Echo, ECG

10.3.2. 2nd Visit (Baseline, 1D, PCI 1M)

1) Efficacy test: CV death, MI, Stroke, BARC bleeding (type 2, 3, or 5), Cardiac death, Death from any cause, Death from vascular cause, Acute MI, Stent thrombosis, Ischemia Driven Revascularization

2) Vital sign
3) Physical examination
4) Adverse event test
5) Dyspnea evaluation
6) Investigational drug prescription
7) Combined medication change
8) Subject suitability test
9) Randomize number given
10) Exploratory test (Optional): Lab test, Cardiac Echo, ECG

10.3.3. 3rd Visit (Treatment, 2M, PCI 3M)

1) Efficacy test: CV death, MI, Stroke, BARC bleeding (type 2, 3, or 5), Cardiac death, Death from any cause, Death from vascular cause, Acute MI, Stent thrombosis, Ischemia Driven Revascularization
2) Vital sign
3) Physical examination
4) Adverse event test
5) Dyspnea evaluation
6) Investigational drug prescription
7) Adherence Assessment
8) Combined medication change
9) Exploratory test (Optional): Lab test, Cardiac Echo, ECG

10.3.4. 4th Visit (Treatment, 5M, PCI 6M)

1) Efficacy test: CV death, MI, Stroke, BARC bleeding (type 2, 3, or 5), Cardiac death, Death from any cause, Death from vascular cause, Acute MI, Stent thrombosis, Ischemia Driven Revascularization
2) Vital sign
3) Physical examination
4) Adverse event test
5) Dyspnea evaluation
6) Investigational drug prescription
7) Adherence Assessment
8) Combined medication change
9) Exploratory test (Optional): Lab test, Cardiac Echo, ECG

10.3.5. 5th Visit (Treatment, 11M, PCI 12M)

1) Efficacy test: CV death, MI, Stroke, BARC bleeding (type 2, 3, or 5), Cardiac death, Death from any cause, Death from vascular cause, Acute MI, Stent thrombosis, Ischemia Driven Revascularization
2) Vital sign
3) Physical examination
4) Adverse event test
5) Dyspnea evaluation
6) Adherence Assessment
7) Combined medication change
8) Exploratory test (Optional): Lab test, Cardiac Echo, ECG

11. Precautions and Expected Side Effects

11.1. Clopidogrel

1) Warning

Patients with genetic CYP2C19 hypofunction: vs. patients with normal CYP2C19 function, systemic exposure of Clopidogrel's active metabolism is low. This lowers the antiplatelet reactions and generally, increases the occurrence of cardiovascular events post myocardial infarction. Once identified as CYP2C19 hypofunction patient, should consider alternative treatment.

2) Adverse Event

Bleeding, hematological disorders (neutropenia/agranulocytosis etc.), gastrointestinal symptoms, rash and other skin diseases etc.

11.2. Ticagrelor

1) Warning

This drug can cause significant or at times, fatal bleeding as in other antithrombotic. Patients with pathologic active bleeding or intracranial hemorrhage should not be given this drug. Patients should stop taking this drug at least 5~7 days prior to any surgery.
Should suspect bleeding if patients show hypotension after taking this drug post coronary angiography, percutaneous coronary intervention (PCI), coronary artery bypass graft (CABG) or other surgeries. If possible, treat bleeding without discontinuing medication. If Ticagrelor treatment is discontinued, risk of cardiovascular event increases.

2) Adverse Event

Bleeding, dyspnea and headache etc.

12. Withdrawal of consent or Loss of follow-up

All enrolled subjects have the right to withdraw their consent or discontinue participation in the study at any time without penalty or loss of benefits. A withdrawn subject will be treated according to the standards of medical care and will not be replaced. Subjects have the right to withdraw from the study at any time without explaining why and without any consequences. When subject discontinues from the trial, investigators record date of discontinuation, reasons for discontinuation, post-treatment and clinical course together with all the data collected until then in the case report form. If a s subject is withdrawn from the study due to problems related to the study drugs, continued follow-up will be needed for subject safety. Otherwise, no additional data will be collected after the subject withdraws. Subjects will be included in the analyses up to the time when the consent was withdrawn unless requesting no use of their medical records for the study analysis.

Subject lost-to-follow-up should be avoided as much as possible and investigators are urged to do their utmost best to maintain subject follow-up compliance. Continuous attempts throughout the final follow-up period should be made to contact the subject. A subject is not considered lost to follow up until the subject’s final follow-up window has closed.

13. Event adjudication and reporting

All clinical endpoints will require clear, prespecified criteria, and centralized review. These endpoints will be captured during patient interview, supplemented by death certificates; hospital record abstracts and related reports (autopsy, biopsy, diagnostic output). All endpoints will be independently adjudicated by the central event adjudication committee. The Investigator must complete the Case Report Form for each endpoint event. The information provided must be sufficient to allow for independent medical assessment of the event. The designee will contact the Investigator should it be necessary to clarify any information. The Investigator should provide any additional follow-up information regarding the event to sponsor as soon as it becomes available. All events should be followed until resolution or stabilization. The study investigators will be responsible to provide all applicable and available source documentation to the Data Coordinating Center (DCC) of Seoul St. Mary’s Hospital (Seoul, Korea) to allow an independent assessment of these events by the CEC members. From extensive experience, the following approach is proposed. First, all

required documents, reports, hospital records will be identified, made anonymous, and copied to the DCC by clinical staff. Second, the DCC will check to ensure confidentiality and, if required, have the records centrally abstracted onto standard forms by trained DCC staff. Third, centrally prepared forms and documents will be circulated to CEC members for assessment.

14. Statistical Analysis

14.1. General Principal of Statistical Analysis

Information collected from subjects of the present clinical trial are analyzed in two forms: ITT (Intention-To-Treatment) and PP (Per-Protocol)

1) ITT analysis group

The ITT population is defined as all randomized patients at 1 month after AMI, regardless of their adherence with the entry criteria, regardless of treatment they actually received, and regardless of subsequent withdrawal from treatment or deviation from the protocol[19]. Only some specific reasons that might cause an exclusion of a patient from the ITT population:

- No treatment was applied at all
- No data are available after randomization
-

2) PP analysis group

The PP population is the subset of ITT population consisting of all patients who receive and retain the treatment during 12 months after PCI[19]. Some specific reasons that might cause an exclusion of a patient from the PP population:

- Violation of entry criteria including inclusion and exclusion criteria
- Withdrawal of consent
- Concomitant treatment of oral anticoagulant agent (vitamin-K antagonists or novel oral anticoagulants such as dabigatran, rivaroxaban, apixaban, or edoxaban) during the study period
- Poor compliance
  - Conversion from ticagrelor + aspirin to clopidogrel + aspirin during RCT procedure and vice versa
  - Discontinuation of test or control drugs for 7 days or longer

* In the cases of withdrawal of consent, concomitant treatment of oral anticoagulation agent and poor compliance, their data will be used for statistical analyses until such events occur.

3) Missing data handling

- Missing variables will not be imputed for planned analyses, except where otherwise specified.
- The primary endpoint will be based on Kaplan-Meier estimates, which automatically account for censored data.
- For sensitivity, purposes, missing data was imputed the most recent data (Last Observation Carried Forward method).

14.2. Evaluation Standard

14.2.1. Efficacy Test Variable

1) Primary Endpoint

Composite endpoint of MACCE (CV death, MI, or stroke) + BARC bleeding (type 2, 3 or 5) between 1 and 12 months after AMI

2) Main Secondary Endpoints

① BARC bleeding (type 2, 3, or 5) between 1 and 12 months after AMI

② Composite endpoint of MACCE (CV death, MI, or stroke) + BARC bleeding (type 3, 5) between 1 and 12 months after AMI

③ Composite endpoint of MACCE (CV death, MI, or stroke) between 1 and 12 months after AMI

3) Other Secondary Endpoints

① All-cause death between 1 and 12 months after AMI

② CV death between 1 and 12 months after AMI

③ Recurrent MI between 1 and 12 months after AMI

④ Stroke between 1 and 12 months after AMI

⑤ Ischemia Driven Revascularization including PCI or CABG between 1 and 12 months after AMI

⑥ Stent thrombosis (definite or probable) between 1 and 12 months after AMI

14.2.2. Exploratory Test Variable

Lab test, cardiac echo, ECG, genetic test

14.2.3. Safety Test Variable

Adverse event, vital sign, physical examination

14.3. Evaluation Method

14.3.1 Primary Endpoint Analysis

- The non-inferiority test between 1 and 12 months after AMI will be based on the Kaplan-Meier estimates. A 95% two-sided confidence interval will be computed for the difference event rate (clopidogrel + aspirin) – event rate (ticagrelor + aspirin). The clopidogrel group will be judged as non-inferior to the ticagrelor if the upper confidence limit is less than the predetermined non-inferiority margin of 3% (absolute risk difference).
- The hypothesis of non-inferiority test will be based on the difference of proportions. Let r_T denote the true event proportion in the test arm (clopidogrel + aspirin) between 1 and 12 months, and r_C denote the true event proportion in the control arm (ticagrelor + aspirin) between 1 and 12 months. The hypotheses are

$$H_0: r_T - r_C \geq \Delta$$

$$H_A: r_T - r_C < \Delta$$

The$\Delta$is the non-inferiority margin, and is taken to be 0.03. The test will be performed as a one-sided test at alpha=0.05.

The null hypothesis shall be rejected at alpha=0.05 if the one-sided p-value is less than 0.05. When this occurs, the upper limit of the two-sided 95% confidence interval will be less than 3%.

- If the non-inferiority analysis passed the acceptance criterion, a superiority analysis will be performed. Statistical superiority is achieved when the upper limit of the two-sided 95% confidence interval of the risk difference is less than 0%. The type I error for this analysis is protected by the non-inferiority analysis, and no alpha adjustment would be appropriate
- Subgroup analyses will be performed by the primary endpoint categorized by type of AMI (STEMI vs NSTEMI), Subgroup analyses will be performed by the primary endpoint categorized by Type of AMI (STEMI vs NTEMI), Gender, Age (≥75 vs <75), Diabetes, LVEF (≥40% vs <40%), eGFR (≥60 vs <60), type of implanted stents (Xience vs Resolute vs Synergy stents), Bleeding risk according to the ARC criteria (high vs low bleeding risk), CYP2C19 loss-of-function carrier status (carrier vs non-carrier).
- The primary analysis population for primary and secondary endpoints will be the Intention-to-Treat (ITT) population. The primary endpoint analysis will also be performed on the Per Protocol (PP) population as subsequent analysis.

- A primary endpoint analysis stratified by the institutions as a sensitivity analysis. Strata will be divided by the accrual number of institution based on quartiles.

14.3.2 Main Secondary Endpoint Analysis

- The secondary endpoints will be composed of two families. The first family consists of the composite endpoint of MACCE (CV death, MI, stroke) plus BARC bleeding (type 2, 3, or 5). The second family will consist of MACCE plus BARC bleeding (type 3, or 5), MACCE, and BARC bleeding (type 2, 3, or 5). The endpoints from the second family will be tested hierarchically, thereby maintaining the study-wise alpha level. These secondary endpoints will only be tested if both the primary composite endpoint and BARC bleeding are significant at non-inferiority analysis, and superiority analysis. Composite endpoint of MACCE plus BARC bleeding (type 3, or 5) will be tested first, and only if this is significant, the composite endpoint of MACCE only will be tested afterwards. BARC bleeding (type 2, 3, or 5) will be tested only if both of the above endpoints are tested significant.

14.3.3 Exploratory Test Variable Analysis

For continuous data, present technical statistics (mean, standard deviation, median, min. and max. value) on the changes between base & each visits. Based on whether the data follow normal distribution, comparatively verify between the two groups using the independent t-test or Wilcoxon’s rank sum test. Also, for comparison within each group, analyze using the paire d t-test or Wilcoxon’s signed rank test. For categorical data, present technical statistics (fre quency, percentage) and comparatively verify between groups using the chi-square test or Fisher’s exact test.

14.3.4 Additional Analysis

Additional analysis should be run including all occurred events if the drug is given continuously.

14.3.5 Safety Test Variable Analysis

14.3.5.1 Adverse event

Analysis should be conducted for all adverse events occurred during the trial. Summarize adverse event occurrence rate, occurrence rate of specific adverse event causing the follow-up loss and occurrence rate of critical adverse event. Adverse event occurrence rate includes all adverse event occurrence rate and occurrence rate of adverse event related to the test drug.

14.3.5.2 Vital sign, physical examination

For continuous data, present technical statistics (mean, standard deviation, median, min. and max. value) on the changes between base & each visits. Based on whether the data follow normal distribution, comparatively verify between the two groups using the independent t-test or

Wilcoxon's rank sum test. Also, for comparison within each group, analyze using the paired t-test or Wilcoxon's signed rank test. For categorical data, present technical statistics (frequency, percentage) and comparatively verify between groups using the chi-square test or Fisher's exact test.

15 Safety (including side-effects) Evaluation Standard, Method and Reporting

15.1 Safety Related Definitions

15.1.1 Adverse Event (AE)

Adverse event is undesired and unintended signs, symptoms and diseases occurred in subjects given the test drug and does not necessarily require a direct correlation to the test drug. Therefore, it includes undesired and unintended signs (i.e. over the clinically meaningful pathological results), symptoms or diseases during the trial regardless of whether the adverse even is related to the test drug or not.

15.1.2 Adverse Drug Reaction (ADR)

Adverse drug reaction is all undesired and unintended reactions caused by any dose of the test drug and cannot disregard the correlation to the test drug.

15.1.3 Serious adverse Event (SAE)

Serious adverse event/adverse drug reaction indicates the below cases.

1) Expired or high risk of death
2) In need of hospitalization or extended hospitalization. Excluding below.
   - not related to the indication of the trial and has not deteriorated after test drug use a nd on standby or prescheduled treatment for existing symptoms
   - emergency room treatment not applying to the definition of serious adverse event and not causing hospitalization

- hospitalization for the purpose of societal issues or respite care without degeneration of over all conditions

3) Causing permanent disability or hypofunction
4) Fetal malformation or abnormality
5) For meaningful cases requiring medical or surgical intervention to prevent subjects from being endangered or prevent the listed results from occurring

15.2 Safety Evaluation Method

15.2.1 Intensity of the Adverse Event

Investigator evaluates the intensity of the adverse event or serious adverse event occurred during the test period. This evaluation should be based on the investigator's clinical judgment.

Intensity of the adverse events and serious adverse events recorded in the case report form should refer to the WHO guideline and adverse events not presented should follow the below standard.

1) Grade1 (mild symptom)
   Adverse events causing temporary or mild inconvenience and does not require treatment. Normal life (function) of subject is not much hindered and activity not limited.
2) Grdae2 (moderate)
   Adverse events from mild to moderate limits on activity. Normal life (function) is considerably hindered, requiring others' help. Treatment may be needed and once recovering from treatment, treatment may not be needed.
3) Grade3 (severe)
   Adverse events with severe limitations on activities, mostly requiring others' assistance. If medical treatment is needed, may require hospitalization.

15.2.2 Correlation of Adverse Event

Correlation of adverse event or serious adverse event to the test drug should be based on the below guideline.

1) Certain
   Correlation to test drug application/usage is valid and cannot be explained by other drugs, chemicals or current diseases. When discontinuing the test drug, show clinically reasonable reactions. If re-administered, definite pharmaceutically and phenomenologically
2) Probable/likely
   Correlation to test drug application/usage is proper and cannot be explained by other drugs, chemicals or current diseases. When discontinuing the test drug, show clinically reasonable reactions (no information on re-administration)
3) Possible
   Although correlation to test drug application/usage is proper, it can also be explained by other drugs, chemicals or current diseases. If information on discontinuation of the test drug is insufficient or unclear
4) Unlikely

If case is temporary and lacking correlation to test drug application/usage. It can also be explained by other drugs, chemicals or potential diseases.

5) Conditional/unclassified
Require more information for review for proper evaluation.

6) Inaccessible/unclassifiable
When information is insufficient and contradictory to evaluate and cannot supplement or confirm

15.3 Reporting of Adverse Event and Serious adverse Event

Investigator should record all information related to adverse events and serious adverse events such as name of adverse event, date of occurrence, end date, continuation at the time of the final evaluation, intensity, correlation to the study drug, results and treatment in the case report form.

15.4 Safety Reporting

If serious adverse event occurs during the clinical trial, it should be reported regardless of its correlation to the test drug.

1) Investigator

Investigator should report all serious adverse events to the IRB immediately and should hand in a follow-up report with the details. In the report, the investigator should use the subject's identification number instead of the name, social security number and address to protect the subject's personal information.

2) Research Coordinator

Research coordinator should report to the investigator immediately when a serious adverse event occurs. Should also follow-up with a detailed report.

3) IRB

IRB should advise the investigator to take necessary actions if unexpected serious adverse drug reactions or new information come up which could negatively impact the subject's safety and the clinical trial.

4) Serious Adverse Event Reporting

Investigator should report all serious adverse events to the IRB immediately. If it causes death or presents risk of death, the investigator should report within 7 days of acknowledgement and also

hand in a follow-up report within 8 days of its first reporting. For all other serious and unexpected adverse drug reaction, the investigator should report to the IRB within 15 days of acknowledgement. Should perform follow-up research if the subject does not recover from the given serious adverse event after the termination of clinical test.

While all serious adverse events should be reported until the end of the trial, serious adverse events occurring within 30 days from test termination, should report only those the investigator considers to be correlated to the test.

5) Major Adverse Cardiac and Cerebrovascular Events [MACCE] & Bleeding Reporting

Principal investigator or research coordinator in each institution should input in the eCRF within 15 days of acknowledgement once a Major adverse cardiac and cerebrovascular event [MACCE] & bleeding occur.

Coordinator in Seoul St. Mary's Hospital should collect the MACCE & Bleeding Event regularly from the eCRF and for unclear variables, should report to the CEAC (Clinical Event Adjudication Committee) members to receive feedback. Feedback should be reported back to the investigator and coordinators in each institution.

16 Informed Consent

Investigator and research coordinator should provide a copy of the informed consent form or any other documents shared with the subject to the subject or representative. If there are any changes to the consent form or shared documents during the clinical trial, the investigator or coordinator should provide a copy of the revised form or document to the subject or his/her representative.

17 Follow-up Treatment of Subjects after Clinical Trial

Test drugs, ticagrelor and clopidogrel are standard treatment drugs for patients with acute myocardial infraction based on myocardial infraction treatment standards of the American Heart Association and the European Heart Association. In Korea, the Health Insurance Corporation approves taking once of the two drugs for acute myocardial infraction. This research treats one of the two drugs once patients are in their stable period post 1M of myocardial infraction. Although this research is randomized, since there is no superiority proven for one of the drugs, patients are expected to certainly, and randomly choose one of drug bearing the side-effects. This research does not apply to the victim compensation agreement.

Investigator should guide no-response or lost to follow-up subjects to get appropriate treatment and for subjects who finished the test, but experienced low efficacy of treatment, switch to other treatment.

If serious adverse event due to the test drug occurs or the disease deteriorate during or after the clinical trial, should receive consultation or treatment anytime and will provide appropriate measures in the emergency room or clinic.

18 Subject Safety and Protective Measures

18.1 Subject Safety and Protective Measures

Switching from Brilinta to Pregrel has no fixed guideline, but is a possible treatment based on the investigator.

According to the guideline of the American Heart Association, acute myocardial infarction patients must take one of the three P2Y12 inhibitors (Clopidogrel 75 mg daily, Prasugrel 10 mg daily, Ticagrelor 90 mg twice daily) after drug emission stent implantation for 1 year, but there is no guideline as to which drug to take as a priority.[11].

According to the research switching to clopidogrel from prasugrel among acute coronary syndrome patients, the effect of platelet inhibition is significantly higher in Brilinta vs. Pregrel[10].

However, Pregrel has been used worldwide prior to the introduction of Brilinta and is currently being used. Pregrel has no limitations of use as it has lower antiplatelet inhibition rate vs. Brilinta, but has sufficient level of platelet inhibition to show effects of treatment.

On the contrary, as the risk of bleeding can be higher for Brilinta with its strong antiplatelet inhibition, this research aims to evaluate the efficacy and safety of the two drugs.

Test drugs are already in-market and the investigator should be fully familiar with the indicated side-effects and precautions in the protocol. In case there are any serious adverse events during the test, the investigator should terminate the clinical trial for the subject, take appropriate measures and immediately inform the IRB.

18.2 Confidentiality

All personal information will be kept confidential under relevant laws and regulations and will not be disclosed to the public. Subject name will not be disclosed to the sponsor and will be indicated only as subject number and initials in the case report form. If diagnostics test result documents has subject’s name, it should be deleted before the copy is shared with the subject. Data recorded in the computer should be kept under the local data protection act. Should notify subject with written document that subject’s medical records may be under due diligence by the staff of the sponsor, IRB or relevant government officials to verify the accuracy. Also, written notification must be given that personal information required for the due diligence will be kept in strict confidentiality under the data protection act. Even after the results are published, information that can be used to identify the subject will be kept confidential.

19 Requirements for Scientific Clinical trial

19.1 Protocol Deviation

Changes that could impact how and what we can get from the clinical trial, including changes in the objective, study design, subject group, sample size estimation and process or changes that can impact the safety of the subject require official change of protocol. These types of deviations must be approved by the IRB prior to the change.

19.2 Record Retention

Investigator should transfer the documents and information to the person in charge of record keeping in the clinical trial institution for 3 years after the closing of the test, unless otherwise specified in other legislations. However, this period can be extended once the head of the Ministry of Food and Drug Safety orders or the sponsor decides necessary. The clinical trial institution should implement back-up plans so that the information is not damaged or missing before the given date.

19.3 Clinical Trial Institution Monitoring

Sponsor or the authorized Clinical Research Organization (CRO) should guarantee that the subjects' human rights, safety and welfare are protected, the test is being conducted appropriately based on the current protocol and GCP, the reported test information are accurate and complete and the relevant documents can be verified. Sponsor has the responsibility to appoint a test monitor for proper monitoring and the monitoring should be conducted based on the monitoring protocol.

19.4 Investigator Responsibility

1) Clinical trial Record and Documents

Investigator should ensure all test related communications, subject records, consent forms, test drug usage records, copy of the case report form are retained. These documents should also be ensured not to be damaged or missing during the record keeping period. However, after the study report is finalized and published (once fact-finding research is completed if required by the head of the Ministry of Food and Drug Safety, documents should be transferred to those in charge of record retention.

2) Protocol Deviation

For major process/protocol changes during the clinical trial -excluding the minor administrative ones

or those not impacting subject's safety- the investigator must receive pre-approval from the IRB.

3) Record Disclosure

Individual medical information obtained from the test is considered confidential and should not be disclosed to any 3rd party other than those with rights to the related information. However, it may be shared with the subject's attending physician or other medical personnel with the responsibility of the subject's welfare. Also, information obtained from this test may be disclosed to the IRB and the Ministry of Food and Drug Safety for due diligence.

20 Study organization

20.1 Steering Committee

The Steering Committee, composed of the chairperson (CI) and the principal investigators of the main participating centers, will approve the trial design, protocol and amendments issued to the Data and Safety Monitoring Board (DSMB) and the clinical sites. This committee will also be responsible for reviewing the final results, determining the methods of presentation and publication, and selection of secondary projects and publications

20.2 Data Safety Monitoring Board (DSMB)

An independent DSMB will monitor the study data on a periodic basis to evaluate interim results during the trial and determine reporting and stopping rules as specified in the DSMB charter and data monitoring plan. The data to be reviewed will consist of adjudicated and non-adjudicated major adverse cardiovascular events, bleeding, and other serious adverse events and their incidence in order to identify potential safety issues. Based on the safety data, the DSMB may recommend modifications to the protocol, suspension or termination of the trial, and advise the Executive Committee. All final decisions regarding trial modifications rest with the Steering Committee. The DSMB committee will review the safety data from this study and make recommendations based on safety analyses, protocol deviation, and follow-up case reports. Scheduled DSMB meetings will discuss safety or compliance issues and will provide advice on modifying or stopping the study as needed. Additionally, the DSMB may call a meeting at any time if there is reason to suspect that safety is an issue. Members will not be among those who directly control the sponsor of this study. Members will not have any affiliation with the core laboratories, or be an Investigator of the trial. The composition of the DSMB will include at least two clinicians with expertise in interventional cardiology and one statistician with expertise in medical statistics and clinical trial. The DSMB will

function in accordance with applicable regulatory guidelines. The DSMB chairperson will notify data coordinating center (DCC) of any safety or compliance issues. The DSMB will help to conduct the trial appropriately by reviewing and reporting the cumulative investigational data for accuracy and completeness, ensuring protocol compliance. The DSMB will develop a consensus understanding of all trial endpoints and definitions used in the event adjudication process.

20.3 Clinical Events Adjudication Committee

The Clinical Events Adjudication Committee (CEAC) is made up of interventional cardiologists who are not participants in the study. The CEC is charged with the development of specific criteria used for the categorization of clinical events in the study which are based on the protocol. At the onset of the trial, the Clinical Events Committee will establish explicit rules outlining the minimum amount of date required, and the algorithm followed in order to classify a clinical event. All members of the CEAC will be blinded to the primary results of the trial. The CEAC will meet regularly to review and adjudicate all clinical events in which the required minimum data is available. The Committee will also review and rule on all clinical events that occur throughout the trial.

20.4 Data Coordination

Data coordination will be performed by the Clinical Research Center in Seoul St. Mary’s Hospital.

21 References

1. Valgimigli M, Bueno H, Byrne RA, Collet JP, Costa F, Jeppsson A, Jüni P, Kastrati A, Kolh P, Mauri L, Montalescot G, Neumann FJ, Petricevic M, Roffi M, Steg PG, Windecker S, Zamorano JL, Levine GN; 2017 ESC focused update on dual antiplatelet therapy in coronary artery disease developed in collaboration with EACTS: The Task Force for dual antiplatelet therapy in coronary artery disease of the European Society of Cardiology (ESC) and of the European Association for Cardio-Thoracic Surgery (EACTS). Eur Heart J. 2018;39(3):213-60.
2. Wallentin L, Becker RC, Budaj A, Cannon CP, Emanuelsson H, Held C, Horrow J, Husted S, James S, Katus H, Mahaffey KW, Scirica BM, Skene A, Steg PG, Storey RF, Harrington RA; PLATO Investigators, Freij A, Thorsén M. Ticagrelor versus clopidogrel in patients with acute coronary syndromes. N Engl J Med. 2009;361(11):1045-57.
3. Wiviott SD, Braunwald E, McCabe CH, Montalescot G, Ruzyllo W, Gottlieb S, Neumann FJ, Ardissino D, De Servi S, Murphy SA, Riesmeyer J, Weerakkody G, Gibson CM, Antman EM; TRITON-TIMI 38 Investigators. Prasugrel versus clopidogrel in patients with acute coro

nary syndromes. N Engl J Med. 2007;357(20):2001-15.

4. Sibbing D, Aradi D, Jacobshagen C, Gross L, Trenk D, Geisler T, Orban M, Gori T, Hadamitzky M, Merkely B, Kiss RG, Komócsi A, Dézsi CA, Thalmeier A, Löw A, Holdt L, Teupser D, Ince H, Felix SB, Parma R, Malek L, Horstkotte J, Baylacher M, Schwinger R, Rieber J, Mudra H, Hausleiter J, Huber K, Neumann FJ, Koltowski L, Huczek Z, Mehilli J, Massberg S; TROPICAL-ACS Investigators. A randomised trial on platelet function-guided de-escalation of antiplatelet treatment in ACS patients undergoing PCI. Rationale and design of the Testing Responsiveness to Platelet Inhibition on Chronic Antiplatelet Treatment for Acute Coronary Syndromes (TROPICAL-ACS) Trial. Thromb Haemost. 2017;117(1):188-95.

5. Velders MA, Abtan J, Angiolillo DJ, Ardissino D, Harrington RA, Hellkamp A, Himmelmann A, Husted S, Katus HA, Meier B, Schulte PJ, Storey RF, Wallentin L, Gabriel Steg P, James SK; PLATO Investigators. Safety and efficacy of ticagrelor and clopidogrel in primary percutaneous coronary intervention. Heart. 2016;102(8):617-25.

6. Morrow DA, Wiviott SD, White HD, Nicolau JC, Bramucci E, Murphy SA, Bonaca MP, Ruff CT, Scirica BM, McCabe CH, Antman EM, Braunwald E. Effect of the novel thienopyridine prasugrel compared with clopidogrel on spontaneous and procedural myocardial infarction in the Trial to Assess Improvement in Therapeutic Outcomes by Optimizing Platelet Inhibition with Prasugrel-Thrombolysis in Myocardial Infarction 38: an application of the classification system from the universal definition of myocardial infarction. Circulation. 2009;119(21):2758-64.

7. Becker RC, Bassand JP, Budaj A, Wojdyla DM, James SK, Cornel JH, French J, Held C, Horrow J, Husted S, Lopez-Sendon J, Lassila R, Mahaffey KW, Storey RF, Harrington RA, Wallentin L. Bleeding complications with the P2Y12 receptor antagonists clopidogrel and ticagrelor in the PLATelet inhibition and patient Outcomes (PLATO) trial. Eur Heart J. 2011;32(23):2933-44.

8. Antman EM, Wiviott SD, Murphy SA, Voitk J, Hasin Y, Widimsky P, Chandna H, Macias W, McCabe CH, Braunwald E. Early and late benefits of prasugrel in patients with acute coronary syndromes undergoing percutaneous coronary intervention: a TRITON-TIMI 38 (TRial to Assess Improvement in Therapeutic Outcomes by Optimizing Platelet InhibitioN with Prasugrel-Thrombolysis In Myocardial Infarction) analysis. J Am Coll Cardiol. 2008;51(21):2028-33.

9. De Luca L, D'Ascenzo F, Musumeci G, Saia F, Parodi G, Varbella F, Marchese A, De Servi S, Berti S, Bolognese L. Incidence and outcome of switching of oral platelet P2Y12 receptor inhibitors in patients with acute coronary syndromes undergoing percutaneous coronary intervention: the SCOPE registry. EuroIntervention. 2017;13(4):459-66.

10. Zettler ME, Peterson ED, McCoy LA, Effron MB, Anstrom KJ, Henry TD, Baker BA, Messenger JC, Cohen DJ, Wang TY; TRANSLATE-ACS Investigators. Switching of adenosine diphosphate receptor inhibitor after hospital discharge among myocardial infarction patients: Insights from the Treatment with Adenosine Diphosphate Receptor Inhibitors: Longitudinal Assessment of Treatment Patterns and Events after Acute Coronary Syndrome (TRANSLATE-ACS) observational study. Am Heart J. 2017;183:62-8.

11. Alexopoulos D, Xanthopoulou I, Deftereos S, Sitafidis G, Kanakakis I, Hamilos M, Angelidis C, Petousis S, Stakos D, Parissis H, Vavouranakis M, Davlouros P, Goudevenos J, Stefanadis C. In-hospital switching of oral P2Y12 inhibitor treatment in patients with acute coronary syndrome undergoing percutaneous coronary intervention: prevalence, predictors and short-term outcome. Am Heart J. 2014;167(1):68-76 e2.

12. Bagai A, Peterson ED, Honeycutt E, Effron MB, Cohen DJ, Goodman SG, Anstrom KJ, Gupta A, Messenger JC, Wang TY. In-hospital switching between adenosine diphosphate receptor inhibitors in patients with acute myocardial infarction treated with percutaneous coronary intervention: Insights into contemporary practice from the TRANSLATE-ACS study. Eur Heart J Acute Cardiovasc Care. 2015;4(6):499-508.

13. Cuisset T, Deharo P, Quilici J, Johnson TW, Deffarges S, Bassez C, Bonnet G, Fourcade L, Mouret JP, Lambert M, Verdier V, Morange PE, Alessi MC, Bonnet JL. Benefit of switching dual antiplatelet therapy after acute coronary syndrome: the TOPIC (timing of platelet inhibition after acute coronary syndrome) randomized study. Eur Heart J. 2017;38(41):3070-8.

14. Sibbing D, Aradi D, Jacobshagen C, Gross L, Trenk D, Geisler T, Orban M, Hadamitzky M, Merkely B, Kiss RG, Komócsi A, Dézsi CA, Holdt L, Felix SB, Parma R, Klopotowski M, Schwinger RHG, Rieber J, Huber K, Neumann FJ, Koltowski L, Mehilli J, Huczek Z, Massberg S; TROPICAL-ACS Investigators. Guided de-escalation of antiplatelet treatment in patients with acute coronary syndrome undergoing percutaneous coronary intervention (TROPICAL-ACS): a randomised, open-label, multicentre trial. Lancet. 2017;390 (10104) :1747-57.

15. Angiolillo DJ, Rollini F, Storey RF, Bhatt DL, James S, Schneider DJ, Sibbing D, So DYF, Trenk D, Alexopoulos D, Gurbel PA, Hochholzer W, De Luca L, Bonello L, Aradi D, Cuisset T, Tantry US, Wang TY, Valgimigli M, Waksman R, Mehran R, Montalescot G, Franchi F, Price MJ. International Expert Consensus on Switching Platelet P2Y12 Receptor-Inhibiting Therapies. Circulation. 2017;136(20):1955-75.

16. Angiolillo DJ. Dual antiplatelet therapy guided by platelet function testing. Lancet. 2017;390(10104):1718-20.

17. Franchi F, Angiolillo DJ. Novel antiplatelet agents in acute coronary syndrome. Nat Rev C

ardiol. 2015;12(1):30-47.

18. ESC Committee for Practice Guidelines (CPG), Jeroen J. Bax (CPG Chairperson) (The Netherlands), Helmut Baumgartner (Germany), Claudio Ceconi (Italy), Veronica Dean (France), Christi Deaton (UK), et al. Third Universal Definition of Myocardial Infarction. J Am Coll Cardiol. 2012 Oct 16;60(16):1581–98.

19. Gwon HC, Hahn JY, Park KW, Song YB, Chae IH, Lim DS, Han KR, Choi JH, Choi SH, Kang HJ, Koo BK, Ahn T, Yoon JH, Jeong MH, Hong TJ, Chung WY, Choi YJ, Hur SH, Kwon HM, Jeon DW, Kim BO, Park SH, Lee NH, Jeon HK, Jang Y, Kim HS. Six-month versus 12-month dual antiplatelet therapy after implantation of drug-eluting stents: the Efficacy of Xience/Promus Versus Cypher to Reduce Late Loss After Stenting (EXCELLENT) randomized, multicenter study. Circulation. 2012;125(3):505-13.

20. Kim BK, Hong MK, Shin DH, Nam CM, Kim JS, Ko YG, Choi D, Kang TS, Park BE, Kang WC, Lee SH, Yoon JH, Hong BK, Kwon HM, Jang Y; RESET Investigators. A new strategy for discontinuation of dual antiplatelet therapy: the RESET Trial (REal Safety and Efficacy of 3-month dual antiplatelet Therapy following Endeavor zotarolimus-eluting stent implantation). J Am Coll Cardiol. 2012;60(15):1340-8.

22. Appendix

Appendix 1. Study Institution, Principal investigator, Sub-investigator and Clinical Research Coordinator

Study Institution		Principal investigator		
		Name	Department	Position
01	The Catholic University of Korea, Seoul ST. Mary's Hospital	Kiyuk Chang	Cardiology	Professor
02	Chonnam National University Hospital,	Myung Ho Jeong	Cardiology	Professor
03	The Catholic University of Korea, Yeouido ST. Mary's Hospital	Chul-Soo Park	Cardiology	Associate Professor
04	The Catholic University of Korea, Uijungbu ST. Mary's Hospital	Woo Seung Shin	Cardiology	Associate Professor
05	The Catholic University of Korea, ST. Paul's Hospital	Dong Bin Kim	Cardiology	Associate Professor
06	The Catholic University of Korea, Bucheon ST. Mary's	Hee-Yeol Kim	Cardiology	Associate Professor

	Hospital			
07	The Catholic University of Korea, Incheon ST. Mary's Hospital	Doo-Soo Chun	Cardiology	Professor
08	The Catholic University of Korea, ST. Vincent's Hospital	Keun Woon Moon	Cardiology	Professor
09	The Catholic University of Korea, Daejeon ST. Mary's Hospital	Mahn-Won Park	Cardiology	Assistant Professor
10	Gangneung Asan Hospital	Sang Shik Jung	Cardiology	Professor
11	Gangwon University Hospital	Byung Ryeul Cho	Cardiology	Professor
12	Kyungsang University Hospital	Jin Shin Ko	Cardiology	Professor
13	Kyunghee University Hospital	Won Kim	Cardiology	Professor
14	Keimyung University Hostpial	Seung Ho Huh	Cardiology	Professor
15	Daegu Catholic University Hospital	Ki Sik Kim	Cardiology	Professor
16	Boramae University	Sang Hyeun Kim	Cardiology	Professor
17	Suncheun ST Carollo General Hospital	Chang Hyeun Cho	Cardiology	Professor
18	Sunchenhyang University Chunan Hospital	Sang Ho Park	Cardiology	Professor
19	Aju University Hospital	Myung Ho Yoon	Cardiology	Professor
20	Youngnam University Hospital	Jong Sun Park	Cardiology	Professor
21	Ulsan University Hospital	Kyung Min Park	Cardiology	Professor
22	Wonju Severance University Hospital	Seung Hwan Lee	Cardiology	Professor
23	Eulju University Hospital	Kyung Tae Chung	Cardiology	Professor
24	Inje University Ilsan Baek Hospital	Joon Hyeung Do	Cardiology	Professor
25	Chungang University Hospital	Sang Wook Kim	Cardiology	Professor
26	Chungju ST Mary's Hospital	Joo Yeoul Baek	Cardiology	Professor
27	Pohang ST Mary's Hospital	Byung Joo Shim	Cardiology	Professor
28	Kangbook Samsung Hospital	Ki Chul Sung	Cardiology	Professor
29	Samsung Changwon Hospital	Ju Hyun Oh	Cardiology	Professor
30	Busan University Hospital	Kwang Soo Cha	Cardiology	Professor
31	Changwon Kyungsang University Hospital	Young Hoon Cho	Cardiology	Professor
32	Inje University Busan Baek Hospital	Jae Sik Jang	Cardiology	Professor

Sub-investigator

Study Institution	Name	Department
The Catholic University of Korea, Seoul ST. Mary's Hospital	Wook Sung Chung	Cardiology
The Catholic University of Korea, Seoul ST. Mary's Hospital	Pum Jun Kim	Cardiology
The Catholic University of Korea, Seoul ST. Mary's Hospital	Hun Jun Park	Cardiology
The Catholic University of Korea, Seoul ST. Mary's Hospital	Ik Jun Choi	Cardiology
The Catholic University of Korea, Seoul ST. Mary's Hospital	Sung Min Yim	Cardiology
The Catholic University of Korea, Seoul ST. Mary's Hospital	Eun Ho Choo	Cardiology
The Catholic University of Korea, Seoul ST. Mary's Hospital	Jin Jin Kim	Cardiology
The Catholic University of Korea, Seoul ST. Mary's Hospital	Min Ok Chang	Cardiology
The Catholic University of Korea, Seoul ST. Mary's Hospital	Jae Kyeung Kim	Cardiology
The Catholic University of Korea, Seoul ST. Mary's Hospital	Dong Kyu Moon	Cardiology
Chonnam National University Hospital,	Youngkeun Ahn	Cardiology
The Catholic University of Korea, Yeouido ST. Mary's Hospital	Keun Ho Park	Cardiology
The Catholic University of Korea, Yeouido ST. Mary's Hospital	Yun-Seok Choi	Cardiology
The Catholic University of Korea, Yeouido ST. Mary's Hospital	Woo Baek Chung	Cardiology
The Catholic University of Korea, Incheon ST. Mary's Hospital	Dong Il Shin	Cardiology
The Catholic University of Korea, Incheon ST. Mary's Hospital	Seok Min Seo	Cardiology
The Catholic University of Korea, Uijeongbu ST. Mary's Hospital	Jong Min Lee	Cardiology
The Catholic University of Korea, Uijeongbu ST. Mary's Hospital	Yoon Seok Koh	Cardiology
The Catholic University of Korea, Uijeongbu ST. Mary's Hospital	Min Kyu Kang	Cardiology
The Catholic University of Korea, ST. Vincent's Hospital	Ki Dong Yoo	Cardiology
The Catholic University of Korea, ST. Vincent's Hospital	Ji Hun Kim	Cardiology
The Catholic University of Korea, ST. Paul's Hospital	Seong Won Chang	Cardiology

The Catholic University of Korea, ST. Paul's Hospital	Byung Hee Hwang	Cardiology
The Catholic University of Korea, Daejeon ST. Mary's Hospital	Chan Joon Kim	Cardiology
The Catholic University of Korea, Daejeon ST. Mary's Hospital	Kyung Min Park	Cardiology
Gangdong Kyung Hee University Hospital	Jin Man Cho	Cardiology
Kyungbook University Hospital	Jang Hoon Lee	Cardiology
Keimyung University Hostpial	Seung Ho Heo	Cardiology
Daegu Catholic University Hospital	Jin Bae Lee	Cardiology
Ulsan University Hospital	Seo Hee Ahn	Cardiology
Eulji University Hospital	Yoo Jung Choi	Cardiology
Eulji University Hospital	Won Ho Kim	Cardiology
Eulji University Hospital	Sang Hyun Park	Cardiology
Inje University Ilsan Baek Hospital	Seung Yoon Lee	Cardiology

STATISTICAL ANALYSIS PLAN

A Prospective, Multicenter, Randomized, Open-label Trial to Compare Efficacy and Safety of Clopidogrel vs Ticagrelor in Stabilized Patients with Acute Myocardial Infarction after Percutaneous Coronary Intervention

Protocol No.: TALOS-AMI

Protocol Version: 7.0

Development date: 2018.06.18

STATISTICAL ANALYSIS PLAN

SIGNITURE PAGE

Protocol No.: TALOS-AMI

Protocol Version: 7.0

Kiyuk Chang, (title)

Printed Name	*signature*	*Date(YYYY/MM/DD)*

Mahn-WonPark, (title)

Printed Name	*signature*	*Date(YYYY/MM/DD)*

Chan Jon Kim, (title)

Printed Name	*signature*	*Date(YYYY/MM/DD)*

Hyeon Woo Lim, (title)

Printed Name	*signature*	*Date(YYYY/MM/DD)*

TABLE OF CONTENTS

LIST OF ABBREVIATIONS AND DEFINITION OF TERMS

1.0 INTRODUCTION
2.0 STUDY OBJECTIVE
3.0 STUDY DESIGN
4.0 ENROLLMENT
5.0 STUDY ENDPOINTS
- **5.1 PRIMARY ENDPOINTS**
- **5.2 SECONDARY ENDPOINTS**

6.0 SUBJECT INCLUSION / EXCLUSION CRITERIA
- **6.1 SUBJECT INCLUSION CRITERIA**
- **6.2 SUBJECT EXCLUSION CRITERIA**

7.0 STUDY PROCEDURES
- **7.1 SCREENING PERIOD**
- **7.2 RANDOMIZATION**

8.0 STATISTICAL ANALYSIS
- **8.1 SAMPLE SIZE CALCULATION**
- **8.2 ANALYSIS POPULATION**
- **8.3 PRIMARY ENDPOINT ANALYSIS**
- **8.4 Main SECONDARY ENDOINT ANALYSIS**
- **8.5 Other Secondary Endpoint Analyses**
- **8.6 Analysis of Subgroups**
- **8.7 GENERAL STATISTICAL METHODOLOGY**
- **8.8 MISSING DATA**

9.0 REFERENCES

1.0 Introduction

In AMI, adequate platelet inhibition is essential to reduce the risk of recurrent thrombotic events. For this reason, dual antiplatelet therapy (DAPT) with aspirin and a P2Y12 inhibitor has become the current mainstay of pharmacological treatment in AMI patients managed with PCI. Although, clopidogrel has an indication for use in AMI[1], potent P2Y12 inhibitors, ticagrelor and prasugrel, compared with clopidogrel have shown significantly improved clinical outcomes in terms of reducing recurrent ischemic events in large randomized trials[2,3]. Thus, current guidelines strongly recommended potent P2Y12 inhibitors for 1 year in AMI patients undergoing PCI[1].

However, along with strong anti-platelet efficacy, a higher risk for bleeding was observed for potent P2Y12 inhibitors compared with clopidogrel in these randomized trials. Intriguingly, benefit due to reduction of ischemic events and harm due to bleeding events predominates at different time points during potent P2Y12 inhibitors treatment[4]. Although the ischemic benefit was consistent throughout the first year after the index event, the benefit of ticagrelor and prasugrel over clopidogrel for reducing thrombotic risk was prominent in the early period after acute coronary syndrome (ACS) when the risk of ischemic complications was highest. In the primary PCI cohort of the PLATO (Platelet Inhibition and Patient Outcomes) trial, ticagrelor showed larger risk reduction for stent thrombosis (ST) during the first 30days of treatment compared with clopidogrel but the difference decreased over time[5]. Similarly, in the TRITON-TIMI (Therapeutic Outcomes by Optimising Platelet InhibitioN with Prasugrel-Thrombolysis In Myocardial Infarction) 38 trial, prasugrel led to a 25% reduction in MI during the first month[6]. On the other hands, the opposite was true for bleeding. Landmark analyses of these two randomized trials revealed that the bleeding risk was similar in the early period of treatment, but there was a larger difference during the chronic period of treatment between potent P2Y12 inhibitors and clopidogrel. Actually most bleeding events predominantly occurred during the maintenance period of treatment[7,8]. As a consequence, to optimize net clinical benefit between early ischemic benefit and late bleeding risks in AMI patients, many physicians have focused on the novel therapeutic strategy of stepwise de-escalation using potent P2Y12 inhibitors only in the acute phase of treatment (during the first 30 days) and using the less potent clopidogrel during the chronic phase of treatment (after the first 30 days).

Despite of the evidence for the consistent efficacy and safety of potent P2Y12 inhibitors with

long-term treatment, de-escalation after ACS is quite common in clinical practice[9-12]. Data have shown that the prevalence of de-escalation during hospitalization ranges from 5% to 14%[10,11] and after discharge ranges from 15% to 28%[12]. However, at present, data from large-scale clinical studies on the topic of de-escalating antiplatelet strategy are very limited and the results of small studies are conflicting[9,13]. Recently, some randomized trials of de-escalation enrolling ACS patients have been reported[13,14]. The randomized, open-label, single-center TOPIC trial (Timing of Optimal Platelet Inhibition After Acute Coronary Syndrome) showed that in patients who have been event free for the first month after an ACS on a combination of aspirin plus a potent P2Y12 inhibitor (ticagrelor or prasugrel), de-escalation to aspirin plus clopidogrel strategy was associated with reduction of bleeding complications without increase in ischemic events[13]. Although this study did not show any differences in ischemic events between groups, play of chance cannot be ruled out given the limited sample size of the trial[15]. The open-label, multicenter TROPICAL-ACS trial (Testing Responsiveness to Platelet Inhibition Chronic Antiplatelet Treatment for ACS) randomized patients with ACS undergoing PCI to either standard treatment with prasugrel for 12 months or a de-escalation regimen (1 week of prasugrel followed by 1 week of clopidogrel and platelet function testing [PFT]–guided maintenance therapy with clopidogrel or prasugrel from day 14 after hospital discharge)[14]. The trial showed that a strategy of PFT-guided de-escalation of antiplatelet treatment was noninferior to standard treatment with prasugrel at 1 year in terms of net clinical benefit. The PFT-guided de-escalation strategy did not show any increase in ischemic events, although there was not a statistically significant reduction in bleeding. However, some experts expressed concerns about a lack of power due to the low number of endpoints events[16]. Furthermore, the routine use of PFT in ACS patients undergoing PCI is limited because it is not widely available in real world clinical practice. And, although prasugrel and ticagrelor have similar levels of platelet inhibition, it might be argued that the study findings cannot be applied to ticagrelor because its pleiotropic effects have been advocated to contribute to its overall benefits[17]. In addition, there are no studies for the de-escalation of antiplatelet treatment enrolling only AMI patients treated by PCI with newer generation DES. In PLATO study, only 60% of population were scheduled for PCI and the patients who underwent PCI received older generation DES.

Therefore, we sought to investigate the efficacy and safety of switching from ticagrelor to clopidogrel in AMI patients with no adverse event during the first month after index PCI with second generation DES.

2.0 Study Objective

The purpose of this trial is to investigate the efficacy and safety of switching from ticagrelor to clopidogrel in stabilized patients with AMI with no adverse events during the first month after an index PCI.

3.0 Study Design

This is a prospective, randomized, open-label, multi-center study. Qualified study patients who conduct screening period for 1 month will be randomized 1:1 to receive either clopidogrel + aspirin as a treatment group or ticagrelor + aspirin as a control one.

4.0 Enrollment

A total of 2590 qualified patients will be enrolled into the study.

5.0 Study Endpoints

5.1 Primary Endpoint

Composite endpoint of MACCE (CV death, MI, stroke) + BARC bleeding (type 2, 3, or 5) between 1 and 12 months after AMI.

5.2 Main Secondary Endpoints

1.BARC bleeding (type 2, 3, or 5) between 1 and 12 months after AMI.

2. Composite endpoint of MACCE (CV death, MI, stroke) + BARC bleeding (type 3, or 5) between 1 and 12 months after AMI.

3.Composite endpoint of MACCE (CV death, MI, stroke) between 1 and 12 months after AMI.

5.3 Other Secondary Endpoints

1. All-cause death between 1 and 12 months after AMI

2. CV death between 1 and 12 months after AMI

3. Recurrent MI between 1 and 12 months after AMI

4. Stroke between 1 and 12 months after AMI

5. Ischemia driven revascularization including PCI or CABG between 1 and 12 months after AMI

6. Stent thrombosis (definite or probable) between 1 and 12 months after AMI

7. Adverse event at12 months after AMI (dyspnea)

Bleeding according the BARC definition and definite or probable stent thrombosis definition are as follows[18].

Table 1. BARC Definition

<table>
<tr><th colspan="3">BARC Definition</th></tr>
<tr><td colspan="2">Type 0</td><td>No bleeding</td></tr>
<tr><td colspan="2">Type 1</td><td>Bleeding that is not actionable and does not cause the patient to seek unscheduled performance of studies, hospitalization, or treatment by a health care professional; may include episodes leading to self-discontinuation of medical therapy by the patient without consulting a health care professional</td></tr>
<tr><td colspan="2">Type 2</td><td>Any overt, actionable sign of haemorrhage (e.g. more bleeding than would be expected for a clinical circumstance; including bleeding found by imaging alone) that does not fit the criteria for Types 3, 4, or 5, but does meet at least one of the following criteria:
(1) requiring non-surgical, medical intervention by a health care professional (2) leading to hospitalization or increased level of care
(3) prompting evaluation.</td></tr>
<tr><td rowspan="2">Type 3</td><td>Type 3a</td><td>Overt bleeding plus hemoglobin drop of 3 to <5*g/dL (provided hemoglobin drop is related to bleed)
Any transfusion with overt bleeding</td></tr>
<tr><td>Type 3b</td><td>Overt bleeding plus hemoglobin drop ≥ 5*g/dL (provided STEMI, NSTEMI drop is related to bleed)
Cardiac tamponade
Bleeding requiring surgical intervention for control (excluding dental/nasal/skin/hemorrhoid)
Bleeding requiring intravenous vasoactive agents</td></tr>
</table>

	Type 3c	Intracranial hemorrhage (does not include microbleeds or hemorrhagic transformation; does include intraspinal)<br>Subcategories; confirmed by autopsy or imaging or LP<br>Intra-ocular bleed compromising vision
Type 4		Coronary artery bypass graft-related bleeding<br>Perioperative intracranial bleeding within 48 hours<br>Reoperation after closure of sternotomy for the purpose of controlling bleeding<br>Transfusion of ≥5 U whole blood or packed red blood cells within a 48 hour period†<br>Chest tube output ≥2 L within a 24-hour period
Type 5	Type 5a	Probable fatal bleeding: no autopsy or imaging confirmation, but clinically suspicious
	Type 5b	Definite fatal bleeding: overt bleeding or autopsy or imaging confirmation

*****:Corrected for transfusion (1 U packed red blood cells or 1 U whole blood =1 g/dL hemoglobin)

† :Cell saver products are not counted.

Table 2 Stent Thrombosis Definition

Stent thrombosis	
Definite*	Angiographic confirmation of stent thrombosis†<br>The presence of a thrombus‡ that originates in the stent or in the segment 5 mm proximal or distal to the stent and presence of at least 1 of the following criteria within a 48-hour time window:<br>Acute onset of ischemic symptoms at rest<br>New ischemic ECG changes that suggest acute ischemia Typical rise and fall in cardiac biomarkers (refer to definition of spontaneous MI)

	Non occlusive thrombus<br>Intracoronary thrombus is defined as a (spheric, ovoid, or irregular) noncalcified filling defect or lucency surrounded by contrast material (on 3 sides or within a coronary stenosis) seen in multiple projections, or persistence of contrast material within the lumen, or a visible embolization of intraluminal material downstream.<br><br>Occlusive thrombus<br>TIMI 0 or TIMI 1 intrastent or proximal to a stent up to the most adjacent proximal side branch or main branch (if originates from the side branch).<br><br>Pathological confirmation of stent thrombosis<br>Evidence of recent thrombus within the stent determined at autopsy or via examination of tissue retrieved following thrombectomy.
Probable	Clinical definition of probable stent thrombosis is considered to have occurred after intracoronary stenting in the following cases:<br>Any unexplained death within the first 30 days§<br>Irrespective of the time after the index procedure, any MI that is related to documented acute ischemia in the territory of the implanted stent without angiographic confirmation of stent thrombosis and in the absence of any other obvious cause

*Definite stent thrombosis is considered to have occurred by either angiographic or pathological confirmation.

†The incidental angiographic documentation of stent occlusion in the absence of clinical signs or symptoms is not considered a confirmed stent thrombosis (silent occlusion).

‡Intracoronary thrombus.

§For studies with ST-elevation MI population, one may consider the exclusion of unexplained death within 30 days as evidence of probable stent thrombosis.

6.0. Subject Inclusion / Exclusion Criteria

6.1 Subject Inclusion Criteria

Subject should meet all of the following criteria.

1. Age ≥ 18 years
2. Patients with AMI (STEMI or NSTEMI) who are administered aspirin and ticagrelor for 30 days after successful PCI with newer-generation drug eluting stents (DES)
3. Female patients with childbearing potential who agree to mandatory pregnancy test and have committed to using adequate contraception
4. Subjects who agree to the study protocol and the schedule of clinical follow-up, and provides informed, written consent, as approved by the appropriate IRB of the respective institution

6.2 Subject Exclusion Criteria

Subject should be excluded if they apply to any of the following criteria.

1. Cardiogenic shock
2. Active internal bleeding, bleeding diathesis, or coagulopathy
3. Gastrointestinal bleeding or genitourinary bleeding, hemoptysis, or vitreous hemorrhage within 2 months
4. Major surgery within 6 weeks
5. History of intracranial bleeding, intracranial neoplasm, intracranial arteriovenous malformation, or intracranial aneurysm
6. Anemia (hemoglobin < 10 g/dL) or platelet count of less than 100,000/mm^3 at the time of screening
7. Concomitant treatment with oral anticoagulant agent (vitamin-K antagonists or novel oral anticoagulants such as dabigatran, rivaroxaban, apixaban, or edoxaban)
8. Daily treatment with non-steroidal anti-inflammatory drug (NSAIDs) or cyclooxygenase-2 inhibitors
9. Malignancy or life expectancy of less than one year
10. Moderate or severe hepatic dysfunction (Child Pugh B or C)

11. Symptomatic patients with sinus bradycardia (sick sinus syndrome) or atrioventricular (AV) block (AV block grade II or III, bradycardia-induced syncope; except for patients implanted with permanent pacemaker)
12. Symptomatic patients with chronic obstructive pulmonary disease (Medical research council grade ≥3)
13. Intolerance of or allergy to aspirin, ticagrelor or clopidogrel
14. Subjects who are under renal replacement therapy due to end-stage renal disease or who have history of kidney transplantation
15. Galactose intolerance, lactase insufficiency or glucose-galactose malabsorption
16. Subjects who are actively participating in another clinical trial with 3 months of randomization (except for observational study)
17. Pregnant and/or lactating women
18. Subjects considered unsuitable for this study by the investigator

7.0 Study Procedure

7.1 Screening period

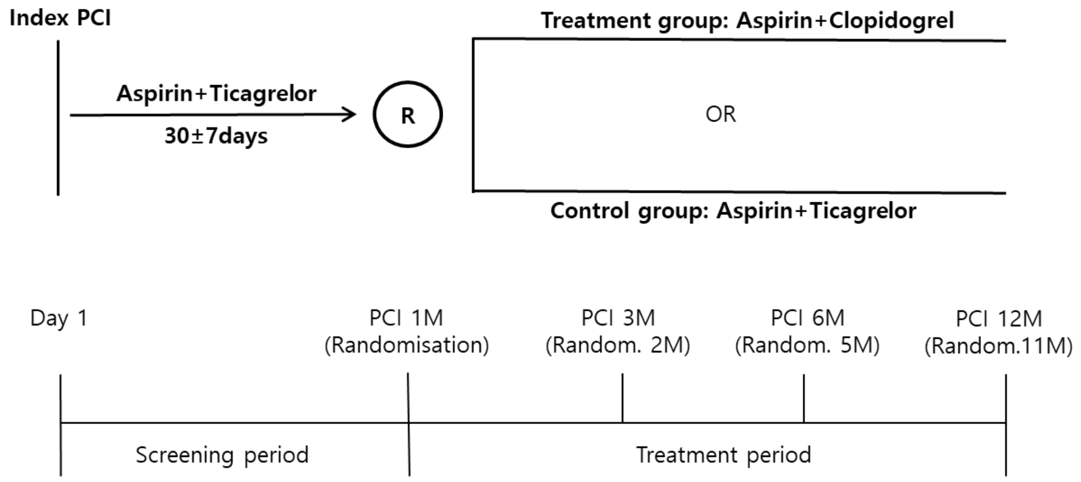

To conduct screening AMI patients based on the inclusion/exclusion criteria who (1) have been treated with ticagrelor + aspirin at least 30 ± 7 days after an index PCI, (2) received full explanation of the study details, (3) given written consent.

7.2 Randomization

Randomization will occur centrally. To randomize a patient, the investigative site will enter the subject into the designated electronic system and obtain the treatment assignment (clopidogrel + aspirin or ticagrelor + aspirin) in a 1:1 ratio. At 1 month visit after AMI, eligible subjects were assigned to each treatment group following an access to the interactive web-based response system (IWRS, Medical Excellence Inc., Seoul, Korea) by the investigator or designee. Randomization sequence was created by an independent statistician using SAS 9.3 (SAS Institute Inc. Cary, NC, USA) statistical software and was stratified by study center and type of AMI (STEMI or NSTEMI) and with a 1:1 allocation using hidden random block size.

8.0 Statistical Analysis

8.1 Sample Size Calculation

The present study is designed to show non-inferiority of the treatment group with aspirin plus clopidogrel versus the control group with aspirin plus ticagrelor. Sample size is based on the combined occurrence rate of ischemic and bleeding events between 1 and 12 months after AMI. According to the PLATO investigators, the event rate of primary efficacy endpoint including CV death, MI or stroke was 5.28% in the ticagrelor group and 6.60% in the clopidogrel group between 1and 12 months after the index event[2].In the meantime, since there were no reported data on the bleeding event rate associated with ticagrelor from 1 to 12months after AMI, especially BARC bleeding rate at the time of the present study design, we assumed the event rate of BARC 2, 3 and 5 bleeding from the event rates of non-CABG related PLATO major or minor bleeding during a year of ticagrelor therapy (8.7%) and non-CABG related major bleeding of first 30 days (2.47%) and after 30 days (2.17%) in the PLATO trial. For the event rate of BARC 2, 3 and 5 bleeding associated with clopidogrel from 1 to 12 months after AMI, the event rate was assumed from the event rates of non-CABG related PLATO major or minor bleeding during a year of clopidogrel therapy (7.0%) and non-CABG related major bleeding of first 30 days (2.21%) and after 30 days (1.65%) in the clopidogrel group of the PLATO trial[7].

After applying mathematical formula, the estimated BARC 2, 3 and 5 bleeding would be 4.07% in the ticagrelor group and 2.99% in the clopidogrel group. Thus, the expected event

rate of the primary endpoint from 1 to12 months after index PCI was 9.35% (ischemic event of 5.28% + bleeding event of 4.07%) in the ticagrelor group and 9.59% (ischemic event of 6.6% + bleeding event of 2.99%) in the clopidogrel group. We chose the non-inferiority margin in accordance with clinical judgment and other relevant studies with a non-inferiority design at the present study design. The non-inferiority margin of two contemporary trials of antiplatelet treatment after PCI that were available up to that time was equivalent to a 40% increase in the expected event rate[18, 19]. The steering committee decided that the non-inferiority margin in our study should be less than a 40% increase compared to the expected event rate of the control group. After considering clinically acceptable relevance and the feasibility of study recruitment, we finally selected the non-inferiority margin of 3.0%, which was equivalent to a 32% increase in the expected event rate. Sample size calculations (PASS 13, NCSS, LLC, Kaysville, Utah, USA) were performed based on one-sided α of 0.05 and a power of 80 %. To achieve these goals, a total of 2,230 patients were needed. With a loss to follow-up rate of 10%, a total of 2,590 (1,295 patients in each group) patients were required.

8.2 Analysis population

The Intent to Treat (ITT) Population

The ITT population is defined as all randomized patients at 1 month after AMI, regardless of their adherence with the entry criteria, regardless of treatment they actually received, and regardless of subsequent withdrawal from treatment or deviation from the protocol[19]. Only some specific reasons that might cause an exclusion of a patient from the ITT population:

- No treatment was applied at all
- No data are available after randomization

The Per Protocol (PP) Population

The PP population is the subset of ITT population consisting of all patients who receive and retain the treatment during 12 months after PCI[19]. Some specific reasons that might cause an exclusion of a patient from the PP population:

- Violation of entry criteria including inclusion and exclusion criteria
- Withdrawal of consent

- Concomitant treatment of oral anticoagulant agent (vitamin-K antagonists or novel oral anticoagulants such as dabigatran, rivaroxaban, apixaban, or edoxaban) during the study period
- Poor compliance
  - Conversion from ticagrelor + aspirin to clopidogrel + aspirin during RCT procedure and vice versa
  - Discontinuation of test or control drugs for 7 days or longer

* In the cases of withdrawal of consent, concomitant treatment of oral anticoagulation agent and poor compliance, their data will be used for statistical analyses until such events occur.

8.3 Primary endpoint analysis

- The non-inferiority test between 1 and 12 months after AMI will be based on the Kaplan-Meier estimates. A 95% two-sided confidence interval will be computed for the difference event rate (clopidogrel + aspirin) – event rate (ticagrelor + aspirin). The clopidogrel group will be judged as non-inferior to the ticagrelor if the upper confidence limit is less than the predetermined non-inferiority margin of 3% (absolute risk difference).
- The hypothesis of non-inferiority test will be based on the difference of proportions. Let r_T denote the true event proportion in the test arm (clopidogrel + aspirin) between 1 and 12 months, and r_C denote the true event proportion in the control arm (ticagrelor + aspirin) between 1 and 12 months. The hypotheses are

$$H_0: r_T - r_C \geq \Delta$$

$$H_A: r_T - r_C < \Delta$$

  The Δ is the non-inferiority margin, and is taken to be 0.03. The test will be performed as a one-sided test at alpha=0.05.

  The null hypothesis shall be rejected at alpha=0.05 if the one-sided p-value is less than 0.05. When this occurs, the upper limit of the two-sided 95% confidence interval will be less than 3%.
- The stratified log-rank test will be performed to test the comparison between time to event distribution Stratification factors will be prior use of STEMI (yes or no).

- Unless otherwise specified, the stratified hazard ratio between two treatment groups along with CI will be obtained by fitting a stratified Cox model with the treatment group variables as unique covariate. Stratification factors will be same as above.
- If the non-inferiority analysis passed the acceptance criterion, a superiority analysis will be performed. Statistical superiority is achieved when the upper limit of the two-sided 95% confidence interval of the risk difference is less than 0%. The type I error for this analysis is protected by the non-inferiority analysis, and no alpha adjustment would be appropriate
- Subgroup analyses will be performed by the primary endpoint categorized by type of AMI (STEMI vs NSTEMI), Subgroup analyses will be performed by the primary endpoint categorized by type of AMI (STEMI vs NTEMI), Gender, Age (≥75 vs <75), Diabetes, LVEF (≥40% vs <40%), eGFR (≥60 vs <60), type of implanted stents (Xience vs Resolute vs Synergy stents), Bleeding risk according to the ARC criteria (high vs low bleeding risk), CYP2C19 loss-of-function carrier status (carrier vs non-carrier).
- The primary analysis population for primary and secondary endpoints will be the Intention-to-Treat (ITT) population. The primary endpoint analysis will also be performed on the Per Protocol (PP) population as subsequent analysis.
- A primary endpoint analysis stratified by the institutions as a sensitivity analysis. Strata will be divided by the accrual number of institution based on quartiles.

8.4 Main Secondary Endpoint Analyses

The secondary endpoints will be composed of two families. The first family consists of the composite endpoint of MACCE (CV death, MI, stroke) plus BARC bleeding (type 2, 3, or 5). The second family will consist of MACCE plus BARC bleeding (type 3, or 5), MACCE, and BARC bleeding (type 2, 3, or 5). The endpoints from the second family will be tested hierarchically, thereby maintaining the study-wise alpha level. These secondary endpoints will only be tested if both the primary composite endpoint and BARC bleeding are significant at non-inferiority analysis, and superiority analysis. Composite endpoint of MACCE plus BARC bleeding (type 3, or 5) will be tested first, and only if this is significant, the composite endpoint of MACCE only will be tested afterwards. BARC bleeding (type 2, 3, or 5) will be tested only if both of the above endpoints are tested significant.

8.5 Other Secondary Endpoint Analyses

The endpoint in this section will be evaluated according to the secondary endpoints described in section 5.2 under the ITT population. Most of secondary analyses were performed by Cox proportional hazard ratio with 95% confidence interval. The following endpoints will be analyzed in using Chi-square test or Fisher's exact test.

- The occurrence of dyspnea at 12 months

8.6 Analysis of Subgroups

The primary and major secondary endpoints will be analyzed in the pre-specified subgroups to evaluate the consistency of results among subgroups of interest. Outcome will be evaluated in the following subgroups:

1) Type of AMI: STEMI vs NTEMI
2) Gender
3) Age: (≥ vs. < median and ≥ vs. <75 years)
4) Diabetes mellitus
5) LVEF: (≥ vs. < median and ≥ vs. <40%)
6) eGFR: ≥60 vs. <60
7) type of implanted stents: Xience vs. Resolute vs. Synergy stent
8) Bleeding risk according to the ARC criteria: high vs. low bleeding risk
9) CYP2C19 loss-of-function allele carrier status: carrier vs. non-carrier

8.7 General Statistical Methodology

- For continuous variables, summary statistics will include means, standard deviations, medians and interquartile range based on normality of variables. Groups will be compared using t-tests or analysis of variance. Where normality violation is observed, Wilcoxon rank-sum test will be performed to compare groups.
- For categorical variables, summary statistics will include numbers and percentages. Group will be compared using Chi-square test or Fisher's exact test.
- Time-dependent variables will be analyzed using the Kaplan-Meier survival curve and group comparison will be used by log-rank statistics including the number of patients-at-risk.

8.8 Missing data

- Missing variables will not be imputed for planned analyses, except where otherwise specified.
- The primary endpoint will be based on Kaplan-Meier estimates, which automatically account for censored data.
- For sensitivity, purposes, missing data was imputed the most recent data (Last Observation Carried Forward method).

9.0. Reference

1. Valgimigli M, Bueno H, Byrne RA, Collet JP, Costa F, Jeppsson A, Jüni P, Kastrati A, Kolh P, Mauri L, Montalescot G, Neumann FJ, Petricevic M, Roffi M, Steg PG, Windecker S, Zamorano JL, Levine GN; 2017 ESC focused update on dual antiplatelet therapy in coronary artery disease developed in collaboration with EACTS: The Task Force for dual antiplatelet therapy in coronary artery disease of the European Society of Cardiology (ESC) and of the European Association for Cardio-Thoracic Surgery (EACTS). Eur Heart J. 2018;39(3):213-60.
2. Wallentin L, Becker RC, Budaj A, Cannon CP, Emanuelsson H, Held C, Horrow J, Husted S, James S, Katus H, Mahaffey KW, Scirica BM, Skene A, Steg PG, Storey RF, Harrington RA; PLATO Investigators, Freij A, Thorsén M. Ticagrelor versus clopidogrel in patients with acute coronary syndromes. N Engl J Med. 2009;361(11):1045-57.
3.Wiviott SD, Braunwald E, McCabe CH, Montalescot G, Ruzyllo W, Gottlieb S, Neumann FJ, Ardissino D, De Servi S, Murphy SA, Riesmeyer J, Weerakkody G, Gibson CM, Antman EM; TRITON-TIMI 38 Investigators. Prasugrel versus clopidogrel in patients with acute coronary syndromes. N Engl J Med. 2007;357(20):2001-15.
4. Sibbing D, Aradi D, Jacobshagen C, Gross L, Trenk D, Geisler T, Orban M, Gori T, Hadamitzky M, Merkely B, Kiss RG, Komócsi A, Dézsi CA, Thalmeier A, Löw A, Holdt L, Teupser D, Ince H, Felix SB, Parma R, Malek L, Horstkotte J, Baylacher M, Schwinger R, Rieber J, Mudra H, Hausleiter J, Huber K, Neumann FJ, Koltowski L, Huczek Z, Mehilli J, Massberg S; TROPICAL-ACS Investigators. A randomised trial on platelet function-guided de-escalation of antiplatelet treatment in ACS patients undergoing PCI. Rationale and design of the Testing Responsiveness to Platelet Inhibition on Chronic Antiplatelet Treatment for Acute Coronary Syndromes (TROPICAL-ACS) Trial. Thromb Haemost. 2017;117(1):188-95.
5. Velders MA, Abtan J, Angiolillo DJ, Ardissino D, Harrington RA, Hellkamp A, Himmelmann

A, Husted S, Katus HA, Meier B, Schulte PJ, Storey RF, Wallentin L, Gabriel Steg P, James SK; PLATO Investigators. Safety and efficacy of ticagrelor and clopidogrel in primary percutaneous coronary intervention. Heart. 2016;102(8):617-25.

6. Morrow DA, Wiviott SD, White HD, Nicolau JC, Bramucci E, Murphy SA, Bonaca MP, Ruff CT, Scirica BM, McCabe CH, Antman EM, Braunwald E. Effect of the novel thienopyridine prasugrel compared with clopidogrel on spontaneous and procedural myocardial infarction in the Trial to Assess Improvement in Therapeutic Outcomes by Optimizing Platelet Inhibition with Prasugrel-Thrombolysis in Myocardial Infarction 38: an application of the classification system from the universal definition of myocardial infarction. Circulation. 2009;119(21):2758-64.

7. Becker RC, Bassand JP, Budaj A, Wojdyla DM, James SK, Cornel JH, French J, Held C, Horrow J, Husted S, Lopez-Sendon J, Lassila R, Mahaffey KW, Storey RF, Harrington RA, Wallentin L. Bleeding complications with the P2Y12 receptor antagonists clopidogrel and ticagrelor in the PLATelet inhibition and patient Outcomes (PLATO) trial. Eur Heart J. 2011;32(23):2933-44.

8. Antman EM, Wiviott SD, Murphy SA, Voitk J, Hasin Y, Widimsky P, Chandna H, Macias W, McCabe CH, Braunwald E. Early and late benefits of prasugrel in patients with acute coronary syndromes undergoing percutaneous coronary intervention: a TRITON-TIMI 38 (TRial to Assess Improvement in Therapeutic Outcomes by Optimizing Platelet InhibitioN with Prasugrel-Thrombolysis In Myocardial Infarction) analysis. J Am Coll Cardiol. 2008;51(21):2028-33.

9. De Luca L, D'Ascenzo F, Musumeci G, Saia F, Parodi G, Varbella F, Marchese A, De Servi S, Berti S, Bolognese L. Incidence and outcome of switching of oral platelet P2Y12 receptor inhibitors in patients with acute coronary syndromes undergoing percutaneous coronary intervention: the SCOPE registry. EuroIntervention. 2017;13(4):459-66.

10. Zettler ME, Peterson ED, McCoy LA, Effron MB, Anstrom KJ, Henry TD, Baker BA, Messenger JC, Cohen DJ, Wang TY; TRANSLATE-ACS Investigators. Switching of adenosine diphosphate receptor inhibitor after hospital discharge among myocardial infarction patients: Insights from the Treatment with Adenosine Diphosphate Receptor Inhibitors: Longitudinal Assessment of Treatment Patterns and Events after Acute Coronary Syndrome (TRANSLATE-ACS) observational study. Am Heart J. 2017;183:62-8.

11. Alexopoulos D, Xanthopoulou I, Deftereos S, Sitafidis G, Kanakakis I, Hamilos M, Angelidis C, Petousis S, Stakos D, Parissis H, Vavouranakis M, Davlouros P, Goudevenos J, Stefanadis C. In-hospital switching of oral P2Y12 inhibitor treatment in patients with acute coronary syndrome undergoing percutaneous coronary intervention: prevalence, predictors and short-term outcome. Am Heart J. 2014;167(1):68-76 e2.

12. Bagai A, Peterson ED, Honeycutt E, Effron MB, Cohen DJ, Goodman SG, Anstrom KJ, Gupta A, Messenger JC, Wang TY. In-hospital switching between adenosine diphosphate receptor inhibitors in patients with acute myocardial infarction treated with percutaneous coronary intervention: Insights into contemporary practice from the TRANSLATE-ACS study. Eur Heart J Acute Cardiovasc Care. 2015;4(6):499-508.

13. Cuisset T, Deharo P, Quilici J, Johnson TW, Deffarges S, Bassez C, Bonnet G, Fourcade L, Mouret JP, Lambert M, Verdier V, Morange PE, Alessi MC, Bonnet JL. Benefit of switching dual antiplatelet therapy after acute coronary syndrome: the TOPIC (timing of platelet inhibition after acute coronary syndrome) randomized study. Eur Heart J. 2017;38(41):3070-8.

14. Sibbing D, Aradi D, Jacobshagen C, Gross L, Trenk D, Geisler T, Orban M, Hadamitzky M, Merkely B, Kiss RG, Komócsi A, Dézsi CA, Holdt L, Felix SB, Parma R, Klopotowski M, Schwinger RHG, Rieber J, Huber K, Neumann FJ, Koltowski L, Mehilli J, Huczek Z, Massberg S; TROPICAL-ACS Investigators. Guided de-escalation of antiplatelet treatment in patients with acute coronary syndrome undergoing percutaneous coronary intervention (TROPICAL-ACS): a randomised, open-label, multicentre trial. Lancet. 2017;390 (10104) :1747-57.

15. Angiolillo DJ, Rollini F, Storey RF, Bhatt DL, James S, Schneider DJ, Sibbing D, So DYF, Trenk D, Alexopoulos D, Gurbel PA, Hochholzer W, De Luca L, Bonello L, Aradi D, Cuisset T, Tantry US, Wang TY, Valgimigli M, Waksman R, Mehran R, Montalescot G, Franchi F, Price MJ. International Expert Consensus on Switching Platelet P2Y12 Receptor-Inhibiting Therapies. Circulation. 2017;136(20):1955-75.

16. Angiolillo DJ. Dual antiplatelet therapy guided by platelet function testing. Lancet. 2017;390(10104):1718-20.

17. Franchi F, Angiolillo DJ. Novel antiplatelet agents in acute coronary syndrome. Nat Rev Cardiol. 2015;12(1):30-47.

18. Mehran R, Rao SV, Bhatt DL, Gibson CM, Caixeta A, Eikelboom J, Kaul S, Wiviott SD, Menon V, Nikolsky E, Serebruany V, Valgimigli M, Vranckx P, Taggart D, Sabik JF, Cutlip DE, Krucoff MW, Ohman EM, Steg PG, White H. Standardized bleeding definitions for cardiovascular clinical trials: a consensus report from the Bleeding Academic Research Consortium. Circulation. 2011;123(23):2736-47.

19. Fisher LD, Dixon DO, Herson J, Frankowski RK, Hearron MS, Peace KE. Intention to treat in clinical trials. In: Peace KE, editor. Statistical issues in drug research and development. New York: Marcel Dekker; 1990. pp. 331–50
