# Supplementary material for: BMI and Deescalation From Ticagrelor to Clopidogrel in Patients With Acute Myocardial Infarction: A Post Hoc Analysis of the TALOS-AMI Trial
Source: JAMA Netw Open. 2025 Feb 27;8(2):e2461916. doi: 10.1001/jamanetworkopen.2024.61916 (PMC11868972; doi:10.1001/jamanetworkopen.2024.61916)
Supplement: Supplement 2. — eTable 1. Procedural Characteristics According to BMI eTable 2. Trend Analysis for BMI as a Continuous Variable eTable 3. Secondary Outcomes eTable 4. Primary Outcomes Based on BMI 25 kg/m2 eFigure. Consort Diagram [file jamanetwopen-e2461916-s002.pdf]

Supplemental Online Content

Bu S, Kim CJ, Lim S, et al. BMI and deescalation from ticagrelor to clopidogrel in patients with acute myocardial infarction: a post hoc analysis of the TALOS-AMI trial. *JAMA Netw Open*. Published online February 27, 2025. doi:101001/jamanetworkopen.2024.61916

eTable 1. Procedural Characteristics According to BMI

eTable 2. Trend Analysis for BMI as a Continuous Variable

eTable 3. Secondary Outcomes

eTable 4. Primary Outcomes Based on BMI 25 kg/m^2

eFigure. Consort Diagram

This supplemental material has been provided by the authors to give readers additional information about their work.

© 2025 Bu S et al. *JAMA Network Open*.

eTable 1. Procedural characteristics according to BMI

	BMI < 28 kg/m² (n = 2344)			BMI ≥ 28 kg/m² (n = 342)			
	De-escalation (n = 1161)	Active control (n = 1183)	P value	De-escalation (n = 184)	Active control (n = 158)	P value	P value (diff)
Access site			0.30			0.14	0.85
Radial	563 (48.5%)	610 (51.6%)		100 (54.4%)	73 (46.5%)		
Femoral	583 (50.2%)	561 (47.4%)		83 (45.1%)	80 (51.0%)		
Both	15 (1.3%)	12 (1.0%)		1 (0.5%)	4 (2.6%)		
GP IIb-IIIa inhibitor	279 (24.0%)	271 (22.9%)	0.52	39 (21.2%)	49 (31.2%)	0.04	0.34
Infarct-related artery							
LM	20 (1.7%)	22 (1.9%)	0.80	1 (0.5%)	2 (1.3%)	0.60	0.22
LAD	592 (51.0%)	568 (48.0%)	0.15	90 (48.9%)	64 (40.5%)	0.12	0.12
LCX	170 (14.6%)	237 (20.0%)	0.00	32 (17.4%)	25 (15.8%)	0.70	0.75
RCA	379 (32.6%)	356 (30.1%)	0.18	60 (32.6%)	66 (41.8%)	0.08	0.04
No. of treated vessels	1.32 ± 0.56	1.33 ± 0.56	0.61	1.30 ± 0.54	1.36 ± 0.63	0.34	0.80
Multivessel treatment							
2 vessels	262 (22.6%)	285 (24.1%)	0.38	37 (20.1%)	36 (22.8%)	0.55	0.41
3 vessels	56 (4.8%)	55 (4.7%)	0.84	15 (8.2%)	6 (3.8%)	0.09	0.26
No. of stents	1.18 ± 0.41	1.17 ± 0.42	0.77	1.23 ± 0.46	1.18 ± 0.40	0.24	0.19
Total stent length, mm	26 (20-38)	26 (20-35)	0.24	28 (20-38)	28 (20-38)	0.82	0.14
Stent diameter, mm	3.0 (2.9-3.5)	3.0 (2.8-3.5)	0.81	3.3 (3.0-3.5)	3.3 (2.8-3.5)	0.44	0.01
OCT	43 (3.8%)	31 (2.7%)	0.14	4 (2.2%)	4 (2.6%)	>.99	0.42
IVUS	286 (24.8%)	270 (23.0%)	0.31	45 (24.6%)	36 (23.5%)	0.82	0.94

Data are presented as n (%) or mean SD.

© 2025 Bu S et al. *JAMA Network Open*.

BMI, body mass index; GP, glycoprotein; IVUS, intravascular ultrasound; LAD, left anterior descending artery; LCX, left circumflex artery; LM, left main coronary artery; No., number; OCT, optical coherence tomography; RCA, right coronary artery; RAD, right coronary artery; No. of stents, number of stents for infarct-related artery

†De-escalation group administered aspirin plus clopidogrel

‡Active control group administered aspirin plus ticagrelor.

© 2025 Bu S et al. *JAMA Network Open*.

eTable 2. Trend analysis for BMI as a continuous variable

	Beta coefficient		Standard Error		P for trend	
	Crude	Adjusted	Crude	Adjusted	Crude	Adjusted
Primary end point	-0.0623	-0.0546	0.0266	0.0272	0.019	0.045
MACE	-0.0820	-0.0584	0.0408	0.0411	0.045	0.156
BARC Bleeding type 2, 3, 5	-0.0526	-0.0511	0.0324	0.0334	0.104	0.126

Adjusted HR is adjusted for age (over> 75 years) and sex.

BARC, Bleeding Academic Research Consortium; HR, hazard ratio; int., interaction; MACE, major adverse cardiovascular events

© 2025 Bu S et al. *JAMA Network Open*.

eTable 3. Secondary outcomes

	BMI < 28 kg/m² (n = 2344)						BMI ≥ 28 kg/m² (n = 342)							
	De-escalation (n = 1161)	Active control (n = 1183)	Crude HR	P value	Adjusted HR	P value	De-escalation (n = 184)	Active control (n = 158)	Crude HR	P value	Adjusted HR	P value	P int.	P int. (adj)
Death														
All-cause	11 (1.0%)	9 (0.8%)	1.25 (0.52–3.01)	0.62	1.28 (0.53–3.09)	0.59	0 (0.0%)	1 (0.6%)	0.29 (0.00–26.43)	0.59	0.25 (0.00–22.73)	0.55	0.41	0.37
Cardiovascular	6 (0.5%)	5 (0.4%)	1.23 (0.37–4.01)	0.74	1.25 (0.38–4.08)	0.72	0 (0.0%)	1 (0.6%)	0.29 (0.00–26.43)	0.59	0.25 (0.00–22.73)	0.55	0.44	0.42
Myocardial infarction														
Any MI	9 (0.8%)	20 (1.7%)	0.46 (0.21–1.01)	0.05	0.46 (0.21–1.01)	0.05	3 (1.6%)	0 (0.0%)	6.05 (0.20–185)	0.30	6.23 (0.19–208)	0.31	0.11	0.12
Spontaneous	7 (0.6%)	14 (1.2%)	0.51 (0.21–1.26)	0.14	0.52 (0.21–1.29)	0.16	2 (1.1%)	0 (0.0%)	4.33 (0.11–179)	0.44	4.41 (0.10–203)	0.45	0.21	0.23
Periprocedural	2 (0.2%)	6 (0.5%)	0.34 (0.07–1.68)	0.19	0.33 (0.07–1.62)	0.17	1 (0.5%)	0 (0.0%)	2.68 (0.03–260)	0.67	2.52 (0.02–271)	0.70	0.99	0.99
Target vessel MI	4 (0.3%)	8 (0.7%)	0.51 (0.15–1.69)	0.27	0.50 (0.15–1.65)	0.25	3 (1.6%)	0 (0.0%)	6.05 (0.20–185)	0.30	6.23 (0.19–208)	0.31	0.99	0.99
Stroke	9 (0.8%)	11 (0.9%)	0.83 (0.35–2.01)	0.68	0.85 (0.35–2.06)	0.73	0 (0.0%)	1 (0.6%)	0.29 (0.00–26.43)	0.59	0.25 (0.00–22.73)	0.55	0.99	0.99
Revascularization														
TVR	12 (1.0%)	15 (1.3%)	0.81 (0.38–1.74)	0.59	0.79 (0.37–1.69)	0.55	5 (2.7%)	2 (1.3%)	2.13 (0.41–10.98)	0.37	2.06 (0.40–10.66)	0.39	0.30	0.28
TLR	9 (0.8%)	9 (0.8%)	1.02 (0.40–2.56)	0.97	0.99 (0.39–2.49)	0.98	5 (2.7%)	0 (0.0%)	9.35 (0.39–223)	0.17	9.12 (0.36–232)	0.18	0.99	0.99
NTVR	15 (1.3%)	20 (1.7%)	0.76 (0.39–1.49)	0.43	0.77 (0.39–1.50)	0.44	3 (1.6%)	2 (1.3%)	1.28 (0.21–7.64)	0.79	1.33 (0.22–7.96)	0.76	0.59	0.61
Stent thrombosis	1 (0.1%)	3 (0.3%)	0.44 (0.05–3.70)	0.45	0.44 (0.05–3.71)	0.45	2 (1.1%)	0 (0.0%)	4.26 (0.10–176)	0.45	4.32 (0.10–197)	0.45	0.99	0.99

adj., adjusted; BMI, body mass index; HR, hazard ratio; int., interaction; MI, myocardial infarction.

© 2025 Bu S et al. *JAMA Network Open*.

eTable 4. Primary outcomes based on BMI 25 Kg/m^2

	BMI < 25 Kg/m^2 (n = 1597)						BMI ≥ 25 Kg/m^2 (n = 1089)							
	De-escalation (n = 793)	Active control (n = 804)	Crude HR	P value	Adjusted HR	P value	De-escalation (n = 552)	Active control (n = 537)	Crude HR	P value	Adjusted HR	P value	P intersect	P intersect (adj)
Primary end point	35 (4.4%)	67 (8.3%)	0.52 (0.35-0.78)	0.00	0.51 (0.34-0.77)	0.00	24 (4.4%)	36 (6.7%)	0.64 (0.38-1.07)	0.09	0.62 (0.37-1.03)	0.07	0.54	0.58
MACE	19 (2.4%)	25 (3.1%)	0.77 (0.42-1.40)	0.39	0.77 (0.42-1.40)	0.39	11 (2.0%)	15 (2.8%)	0.71 (0.33-1.55)	0.39	0.69 (0.32-1.51)	0.35	0.88	0.82
BARC Bleeding														
type 2, 3, 5	20 (2.5%)	46 (5.7%)	0.43 (0.26-0.73)	0.00	0.43 (0.25-0.73)	0.00	16 (2.9%)	25 (4.7%)	0.62 (0.33-1.15)	0.13	0.60 (0.32-1.12)	0.11	0.40	0.42
type 3, 5	9 (1.1%)	20 (2.5%)	0.45 (0.21-1.00)	0.05	0.45 (0.21-1.00)	0.05	6 (1.1%)	8 (1.5%)	0.73 (0.25-2.09)	0.55	0.71 (0.25-2.04)	0.52	0.49	0.51
type 2	15 (1.9%)	31 (3.9%)	0.49 (0.26-0.90)	0.02	0.48 (0.26-0.88)	0.02	12 (2.2%)	19 (3.5%)	0.61 (0.30-1.25)	0.19	0.60 (0.29-1.23)	0.16	0.64	0.65
type 3	9 (1.1%)	20 (2.5%)	0.45 (0.21-1.00)	0.05	0.45 (0.21-1.00)	0.05	6 (1.1%)	8 (1.5%)	0.73 (0.25-2.09)	0.55	0.71 (0.25-2.05)	0.53	0.49	0.51
type 5	1 (0.1%)	0 (0.0%)					0 (0.0%)	0 (0.0%)						

Adjusted HR is adjusted for age (over> 75 years) and sex.

adj., adjusted; BARC, Bleeding Academic Research Consortium; HR, hazard ratio; int., interaction; MACE, major adverse cardiovascular events

© 2025 Bu S et al. *JAMA Network Open*.

eFigure 1. Consort diagram.

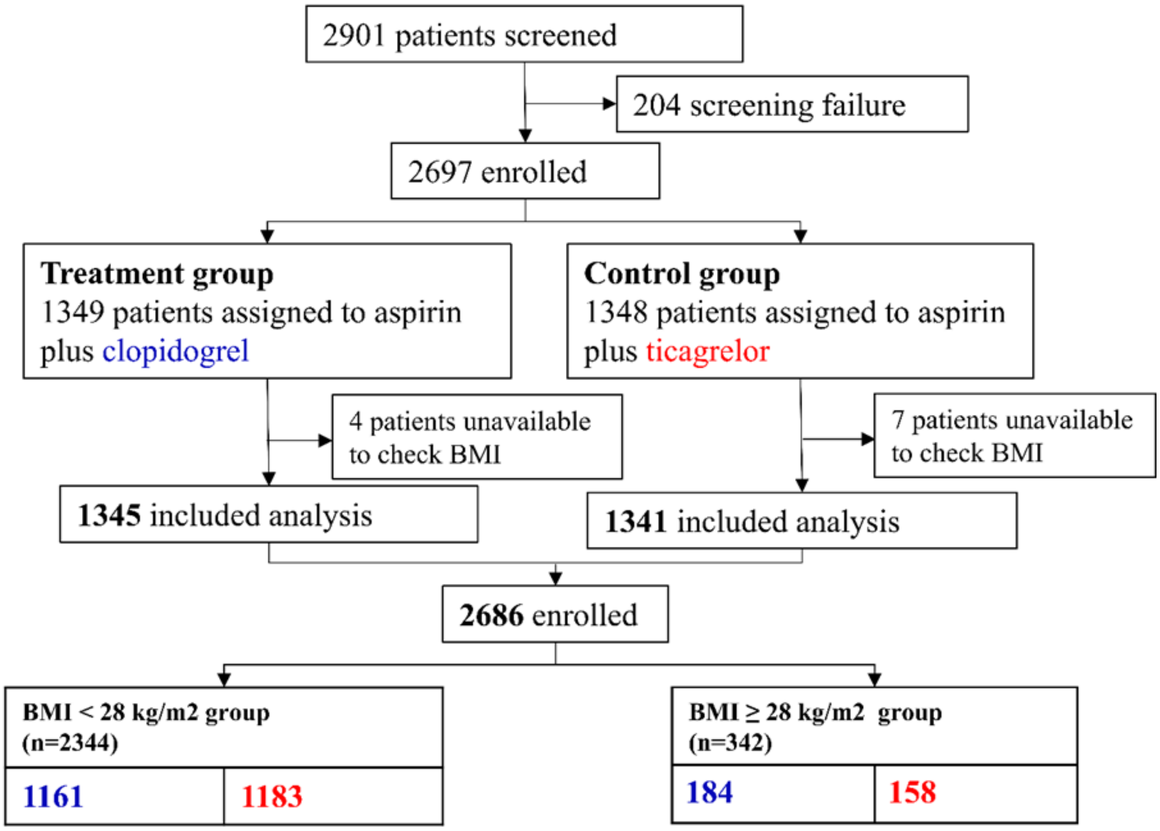

**N, (%)**	**BMI < 28 kg/m2 group (n=2344)**	**BMI ≥ 28 kg/m2 group (n=342)**	**Total (n=2686)**
De-escalation group<br>ASA + CLOPD	1161 (86.3)	184 (13.7)	1345
Active control group<br>ASA + TCGL	1183 (88.2)	158 (11.8)	1341

BMI (in kg/m2) was calculated as weight in kilogram divided by height in meter squared. ASA, aspirin; BMI, body mass index; CLOPD, clopidogrel; TCGL, ticagrelor.

© 2025 Bu S et al. *JAMA Network Open*.
