# Supplementary material for: BMI and Deescalation From Ticagrelor to Clopidogrel in Patients With Acute Myocardial Infarction: A Post Hoc Analysis of the TALOS-AMI Trial
Source: JAMA Netw Open. 2025 Feb 27;8(2):e2461916. doi: 10.1001/jamanetworkopen.2024.61916 (PMC11868972; doi:10.1001/jamanetworkopen.2024.61916)
Supplement: Supplement 3. — Nonauthor Collaborators [file jamanetwopen-e2461916-s003.pdf]

Supplemental Online Content: Nonauthor Collaborators
*First name, last name, and suffix (if applicable) are required and will appear in PubMed.

*Group Name(s): The TALOS-AMI investigators

*First Name and Middle Initial(s)	*Last Name	*Suffix (eg, Jr, III)	Academic Degrees	Institution	Location (city, state/province, country)	Role or Contribution, eg, chair, principal investigator	Group (if more than 1 Group listed in the byline) and/or Subgroup (eg, Steering Committee)
Myung Ho	Jeong		MD, PhD	Chonnam National university hospita	Gwangju, Republic of Kore	PI	Steering Committee
Chul-Soo	Park		MD, PhD	Yeouido St. Mary's hospital	Seoul, Republic of Korea	PI	Steering Committee
Woo Seung	Shin		MD, PhD	Uijeongbu St. Mary's hospital	Uijeongbu-si, Gyeonggi- do	PI	Steering Committee
Dong Bin	Kim		MD, PhD	St. Paul's hospital	Seoul, Republic of Korea	PI	Steering Committee
Sang Shik	Jung		MD, PhD	Gangneung Asan hospital	Gangneung-si, Gangwon- d	PI	Steering Committee
Byung Ryeol	Cho		MD, PhD	Gangwon university hospital	Chuncheon-si, Gangwon- d	PI	Steering Committee
Jin Shin	Ko		MD, PhD	Kyungsang National university hospit	Jinju-si, Gyeonsangnam- do	PI	Steering Committee
Won	Kim		MD, PhD	Kyunghee university hospital	Seoul, Republic of Korea	PI	Steering Committee
Seung Ho	Huh		MD, PhD	Keimyung university hospital	Daegu, Republic of Korea	PI	Steering Committee
Ki Sik	Kim		MD, PhD	Daegu Catholic university hospital	Daegu, Republic of Korea	PI	Steering Committee
Sang Hyeon	Kim		MD, PhD	Boramae hospital	Seoul, Republic of Korea	PI	Steering Committee
Chang Hyeon	Cho		MD, PhD	St. Carollo general hospital	Suncheon-si, Jeollanam- do	PI	Steering Committee
Sang Ho	Park		MD, PhD	Soonchunhyang university Cheonan h	Cheonan-si, Chungcheongn	PI	Steering Committee
Myung Ho	Yoon		MD, PhD	Ajou university hospital	Suwon-si, Gyeonggi-do, Re	PI	Steering Committee
Jong Sun	Park		MD, PhD	Youngnam university hospital	Daegu, Republic of Korea	PI	Steering Committee
Kyung Min	Park		MD, PhD	Ulsan university hospital	Ulsan, Republic of Korea	PI	Steering Committee
Seoung Hwan	Lee		MD, PhD	Wonju Severance hospital	Wonju-si, Gangwon-do, Re	PI	Steering Committee
Kyung Tae	Chung		MD, PhD	Eulji university hospital	Daejeon, Republic of Korea	PI	Steering Committee
Joon Hyeong	Do		MD, PhD	Inje university Ilsan Baek hospital	Ilsan-si, Gyeonggi-do, Repu	PI	Steering Committee
Sang Wook	Kim		MD, PhD	Chungang university hospital	Seoul, Republic of Korea	PI	Steering Committee
Joo Yeol	Baek		MD, PhD	Cheongju St. Mary's hospital	Cheongju-si, Chungcheongl	PI	Steering Committee
Byung Joo	Shim		MD, PhD	Pohang St. Mary's hospital	Pohang-si, Gyeonsangbuk-	PI	Steering Committee
Ki Chul	Sung		MD, PhD	Kangbook Samsung hospital	Seoul, Republic of Korea	PI	Steering Committee
Ju Hyun	Oh		MD, PhD	Samsung Changwon hospital	Changwon-si, Gyeonsangna	PI	Steering Committee
Kwang Soo	Cha		MD, PhD	Busan university hospital	Busan, Republic of Korea	PI	Steering Committee
Young Hoon	Cho		MD, PhD	Changwon Kyunsang university hospi	Changwon-si, Gyeonsangna	PI	Steering Committee
Jae Sik	Jang		MD, PhD	Inje university Busan Baek hospital	Busan, Republic of Korea	PI	Steering Committee
Jin Man	Cho		MD, PhD	Gangdong Kyunghee university hospi	Seoul, Republic of Korea	PI	Steering Committee
Jang Hoon	Lee		MD, PhD	Kyungbook university hospital	Daegu, Republic of Korea	PI	Steering Committee
