# Supplementary material for: BMI and Deescalation From Ticagrelor to Clopidogrel in Patients With Acute Myocardial Infarction: A Post Hoc Analysis of the TALOS-AMI Trial
Source: JAMA Netw Open. 2025 Feb 27;8(2):e2461916. doi: 10.1001/jamanetworkopen.2024.61916 (PMC11868972; doi:10.1001/jamanetworkopen.2024.61916)
Supplement: Supplement 4. — Data Sharing Statement [file jamanetwopen-e2461916-s004.pdf]

Data Sharing Statement

Bu. BMI and Deescalation From Ticagrelor to Clopidogrel in Patients With Acute Myocardial Infarction. *JAMA Netw Open*. Published February 27, 2025.
doi:10.1001/jamanetworkopen.2024.61916

Data
Additional Information: ClinicalTrials.gov identifier: NCT02018055
Data available: No

Additional Information
Explanation for why data not available: The TALOS-AMI trial is planned to continue followup. Until the end of the follow-up, no individual participant data will be available
